# Glucose metabolism controls monocyte homeostasis and migration but has no impact on atherosclerosis development in mice

**DOI:** 10.1038/s41467-024-53267-5

**Published:** 2024-10-19

**Authors:** Alexandre Gallerand, Bastien Dolfi, Marion I. Stunault, Zakariya Caillot, Alexia Castiglione, Axelle Strazzulla, Chuqiao Chen, Gyu Seong Heo, Hannah Luehmann, Flora Batoul, Nathalie Vaillant, Adélie Dumont, Thomas Pilot, Johanna Merlin, Fairouz N. Zair, Jerome Gilleron, Adeline Bertola, Peter Carmeliet, Jesse W. Williams, Rafael J. Arguello, David Masson, David Dombrowicz, Laurent Yvan-Charvet, Denis Doyen, Arvand Haschemi, Yongjian Liu, Rodolphe R. Guinamard, Stoyan Ivanov

**Affiliations:** 1 Université Côte d’Azur, CNRS, LP2M Nice, France; 2 Université Côte d’Azur, INSERM, C3M, Nice, France; 3 Department of Laboratory Medicine, Medical University of Vienna, 1090 Vienna, Austria; 4 Department of Radiology, Washington University School of Medicine, Saint Louis, MO USA; 5 Université Bourgogne Franche-Comté, LNC UMR1231, F-21000 Dijon, France; 6 Laboratory of Angiogenesis and Vascular Metabolism, Center for Cancer Biology (CCB), VIB, Department of Oncology, Leuven Cancer Institute (LKI), KU Leuven, Leuven, 3000 Belgium; 7 Center for Immunology, Department of Integrative Biology and Physiology, University of Minnesota Medical School, Minneapolis, MN USA; 8 Aix Marseille University, CNRS, INSERM, CIML, Centre d’Immunologie de Marseille-Luminy, Marseille, France; 9 Univ.Lille, INSERM, CHU Lille, Institut Pasteur de Lille, U1011-EGID, 59000 Lille, France; 10 Médecine Intensive Réanimation, Hôpital Pasteur, CHU de Nice, Nice, France

**Keywords:** Monocytes and macrophages, Cardiovascular diseases, Chemokines, Metabolism

## Abstract

Monocytes directly contribute to atherosclerosis development by their recruitment to plaques in which they differentiate into macrophages. In the present study, we ask how modulating monocyte glucose metabolism could affect their homeostasis and their impact on atherosclerosis. Here we investigate how circulating metabolites control monocyte behavior in blood, bone marrow and peripheral tissues of mice. We find that serum glucose concentrations correlate with monocyte numbers. In diet-restricted mice, monocytes fail to metabolically reprogram from glycolysis to fatty acid oxidation, leading to reduced monocyte numbers in the blood. Mechanistically, Glut1-dependent glucose metabolism helps maintain CD115 membrane expression on monocytes and their progenitors, and regulates monocyte migratory capacity by modulating CCR2 expression. Results from genetic models and pharmacological inhibitors further depict the relative contribution of different metabolic pathways to the regulation of CD115 and CCR2 expression. Meanwhile, Glut1 inhibition does not impact atherosclerotic plaque development in mouse models despite dramatically reducing blood monocyte numbers, potentially due to the remaining monocytes having increased migratory capacity. Together, these data emphasize the role of glucose uptake and intracellular glucose metabolism in controlling monocyte homeostasis and functions.

## Abstract (web summary)

Monocytes participate in plaque formation, and adapt to metabolic changes to alter their functions. Here the authors show, using genetic mouse models and functional analyses, that Glut1-mediated glucose metabolism is important for regulating monocyte homeostasis and migration, but curiously has no impact on atherosclerotic plaque formation.

## Introduction

Monocytes are innate immune cells generated in the bone marrow compartment^1,2^. Monocytes differentiate from hematopoietic stem cells (HSCs) in a multistep tightly regulated process. In murine blood, two major monocyte populations have been distinguished according to their expression of the marker of unknown function Ly6C^3^. Inflammatory Ly6C^high^ monocytes, also called classical monocytes, highly express the chemokine receptor CCR2 and can infiltrate peripheral tissues to maintain macrophage pool homeostasis^4–6^. Patrolling Ly6C^low^ monocytes, also known as non-classical monocytes, sample and heal blood vessels^7,8^. Counterparts of murine classical and non-classical monocytes are also found in humans and are identified as CD14^+^ CD16^-^ and CD14^-^ CD16^+^, respectively. Both monocyte subsets express CD115 (CSF1R) which binds M-CSF (CSF1) and IL-34, the main cytokines responsible for maintenance of tissue macrophages. The mechanisms controlling CD115 and CCR2 expression on monocytes are yet to be fully understood.

Monocytes play central roles during acute and chronic inflammation^2^. Their recruitment to the site of inflammation and subsequent differentiation into different subsets of tissue macrophages prevent or amplify inflammation depending on the local microenvironment. High blood monocyte counts positively associate with atherosclerosis progression in humans^9,10^. Thus, controlling monocyte numbers and fate decisions is of critical importance during infections and cardio-metabolic diseases. In pre-clinical models, monocytes contribute to atherosclerotic plaque growth after being recruited via chemokine receptors such as CCR2, CCR5 and CX3CR1^11^ and differentiating into macrophages. Mice lacking CSF1 barely form plaques, suggesting a crucial role of the CSF1-CSF1R axis in plaque formation^12^. Controlling monocyte numbers and their plaque recruitment represent attractive therapeutical strategies.

During the past decade, intracellular metabolism emerged as a key regulator of macrophage and dendritic cell (DC) functions^13^. Glucose metabolism controls key macrophage functions including efferocytosis and cytokine release^14–18^. Most recently, it was demonstrated that amino acid metabolism is also crucial to sustaining optimal macrophage efferocytosis^19,20^. However, less is known regarding how metabolism modulates monocyte numbers and functions. Ablation of Glut1, the main glucose transporter, in myeloid cells prevented hyperglycemia-induced increase in blood monocyte counts^16^. A previous study demonstrated that fasting for short (4 h) or longer (20–24 h) periods, induced decreased blood monocyte numbers due to their retention in the bone marrow compartment^21,22^. Importantly, the authors observed that carbohydrate and protein supplementation, but not fatty acids, restored normal blood monocyte numbers^21^. How precisely glucose flux and intracellular rewiring of respective metabolic pathways affect monocyte survival, proliferation and migration is yet to be defined. Thus, a better understating of how metabolism controls monocyte homeostasis might provide new tools to regulate their numbers and functions in the context of metabolic diseases and their cardiovascular complications, such as atherosclerosis. In the present study, we sought to explore how monocyte intracellular metabolism might affect their functions at steady state and during atherosclerosis progression. We found that Glut1-dependent glucose uptake regulated monocyte generation and survival. Interestingly, systemic inhibition of Glut1 did not impact atherosclerotic plaque formation despite a drastic reduction of blood monocyte numbers, as remaining monocytes had an enhanced capacity to migrate to plaques. At a cell-autonomous level, Glut1-dependent glucose uptake regulated CCR2 through glycolysis and CD115 expression through hexosamine biosynthetic pathway-dependent glycosylation.

In the present study, we explore how metabolic cues regulate monocyte biology and report that Glut1-dependent glucose uptake is crucial in maintaining monocyte generation and survival. While monocytes heavily contribute to atherosclerosis development, reducing blood monocyte numbers through Glut1 inhibition does not prevent plaque formation due to increased chemotaxis of few remaining monocytes. Mechanistically, glucose regulates CCR2 expression through glycolysis while the hexosamine biosynthetic pathway regulates CD115 expression in both murine and human monocytes. Our observations thus suggest that targeting monocyte intracellular glucose metabolism, rather than their glucose uptake or systemic glucose levels, could offer opportunities to finely tune monocyte recruitment and fate as macrophages.

## Results

### Fasting and refeeding regulate blood monocyte numbers and their expression of CD115 and CCR2

**Fig. 1:**
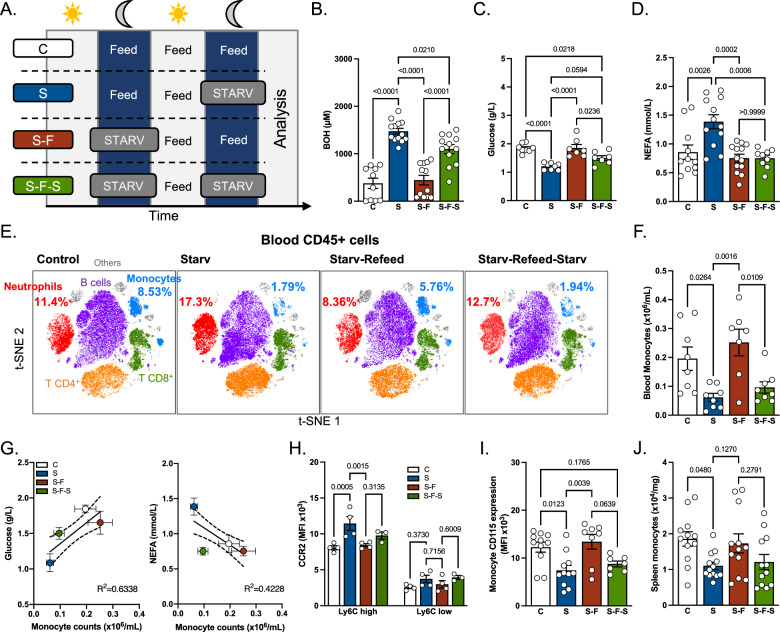
Impact of starvation on peripheral monocytes and neutrophils. **A** Experimental scheme. Age-matched CX3CR1^GFP^ mice were randomly assigned to control (C), starvation (S), starvation-refeeding (S-F) and starvation-refeeding-starvation (S-F-S) groups. For starvation, mice were moved to a clean cage and food was completely removed at ZT10. For refeeding, food was made available again at ZT0 the next day. For each experiment, all four groups were analyzed on the same day at ZT0. **B**–**D** Quantification of serum β-hydroxybutyrate (C *n* = 10, S *n* = 13, S-F n = 12, S-F-S *n* = 12) (**B**), glucose (C *n* = 8, S *n* = 8, S-F *n* = 7, S-F-S *n* = 7) (**C**) and non-esterified fatty acids (C *n* = 11, S *n* = 12, S-F *n* = 13, S-F-S *n* = 9) (**D**). Data pooled from three independent experiments. **E** Dimensional reduction of flow cytometry data to illustrate blood leukocyte subset proportions depending on experimental conditions. **F** Quantification of blood monocyte numbers using flow cytometry (C *n* = 8, S *n* = 8, S-F *n* = 7, S-F-S *n* = 8). Data pooled from two independent experiments. **G** Correlation between blood monocyte numbers and serum glucose or NEFA levels. **H** Quantification of surface CCR2 expression on blood Ly6C^high^ and Ly6C^low^ monocytes (*n* = 4 mice per condition). Data representative of two independent experiments. **I** Measurement of surface CD115 expression on blood monocytes (C *n* = 11, S *n* = 11, S-F *n* = 8, S-F-S *n* = 7). Data pooled from two independent experiments. **J** Quantification of spleen monocytes (C *n* = 12, S *n* = 13, S-F *n* = 12, S-F-S *n* = 12). Data pooled from 3 independent experiments. One-way ANOVA with Tukey’s multiple comparison tests were used for statistical analysis in (**B**–**D**), (**F**), and (**I**, **J**). A two-way ANOVA with Tukey’s multiple comparison test was used for statistical analysis in (**H**). Data are presented as mean values +/− SEM. See also Fig. S1. Source data are provided as a Source data file.

We sought to determine the influence of metabolic fluctuations on monocyte dynamics in blood and peripheral tissues. An elegant study demonstrated that food deprivation provoked a decrease in peripheral blood monocyte numbers due to their bone marrow retention^21^. We thus designed a protocol of overnight starvation followed by refeeding and a second round of starvation (Fig. 1A) in order to explore whether monocytes would respond similarly to repeated metabolic challenge. In this setting, the first round of starvation induced a massive generation of ketone bodies (Fig. 1B). β-hydroxybutyrate (BOH) and acetoacetate (AcAc) levels were both increased in starved animals in comparison to fed controls (Figs. 1B and S1A). Refeeding mice with chow diet diminished ketone body serum concentration to basal levels (Figs. 1B and S1A). BOH mobilization was dampened during the second round of starvation (Fig. 1B). Carnitine serum concentration decreased after the first starvation and was restored following refeeding. However, the second starvation failed to diminish serum carnitine concentration (Fig. S1B). Glucose levels were significantly lower in mice starved for 12 h (Fig. 1C). Non-esterified fatty acids (NEFA) levels increased following the first starvation but were not modulated by the second starvation cycle (Fig. 1D). Triglyceride serum concentration diminished after the first round of starvation and was further decreased following the second starvation cycle (Fig. S1C). Therefore, starving mice induced a specific metabolic reprogramming that translated into a modified serum metabolite profile following the second round of starvation. As expected, glucose levels were decreased after starvation while levels of NEFAs and ketone bodies were increased.

We next sought to determine whether fluctuations in serum metabolites induced a specific blood immune cell pattern. Analysis of blood leukocyte content (Figs. 1E, S2A and S1D) revealed that monocyte numbers strongly diminished following starvation (Fig. 1E, F). Refeeding rapidly restored blood monocyte counts while the second starvation triggered monocyte decrease, to an extent similar to the first round of starvation (Fig. 1E, F). Detailed analysis of monocyte subsets revealed that those fluctuations mainly occurred in Ly6C^hi^ monocytes, while effects on Ly6C^low^ monocytes were mostly not statistically significant (Fig. S1E). Neutrophils displayed the opposite pattern, characterized by increased numbers after starvation (Figs. 1E and S1F). Importantly, blood monocyte numbers strongly correlated with plasma glucose concentration but not NEFAs (Fig. 1G). These data suggested that monocytes might rely on glucose for their maintenance.

Unexpectedly, the remaining Ly6C^high^, but not Ly6C^low^, blood monocytes in starved mice displayed a higher membrane CCR2 expression (Fig. 1H). This expression pattern seemed restricted to CCR2 since the membrane expression of CX3CR1 and CXCR4, two other chemotactic receptors expressed by monocytes, were not affected by starvation and refeeding (Fig. S1G,H). Furthermore, starvation reduced CD115 (CSF1R) expression on blood monocytes and refeeding rapidly restored its basal expression (Fig. 1I). We next investigated whether monocyte numbers were regulated in a similar way in the spleen, a well-established reservoir for monocytes. Spleen weight considerably decreased following the first starvation (Fig. S1I). After refeeding, the spleen regained its initial weight which decreased rapidly following the second starvation (Fig. S1I). These data demonstrated that the spleen swiftly adapted to the host’s metabolic status by modulating its content. Splenic monocytes showed oscillations similar to their blood counterparts (Figs. 1J and S2B). Indeed, there was starvation-induced decrease in splenic monocyte numbers, which recovered back to steady-state level following refeeding (Fig. 1J). As previously reported^21^, serum levels of CCL2, the main ligand for CCR2, attracting monocytes from bone marrow and into tissues, were also modulated by the host’s metabolic status. Starvation tended to decrease CCL2 serum concentration while refeeding restored the steady-state levels (Fig. S1J). CCL2 levels also diminished significantly after the second starvation (Fig. S1J). Whether this is due to decreased secretion or increased consumption, or elimination remains to be established.

### Glut1-dependent glucose uptake regulates monocyte survival and CD115 expression

Food deprivation was shown to trigger reduction of blood monocyte numbers due to their bone marrow retention^21^. In our experimental protocol, we observed that starvation and refeeding did not influence bone marrow mature monocyte and neutrophil numbers (Fig. S1K). Hematopoiesis and myelopoiesis seemed unaffected in these experimental conditions as hematopoietic stem cells (Lin^-^ Sca1^+^ cKit^+^, named LSK), GMP (granulocyte monocyte progenitor) and CMP (common myeloid progenitor) numbers remained similar in starved and refed mice (Fig. S1L). These data suggested that loss of blood monocytes during starvation was likely not linked to altered myelopoiesis or their bone marrow retention. Rather, circulating blood monocyte numbers appeared regulated by glucose availability.

**Fig. 2:**
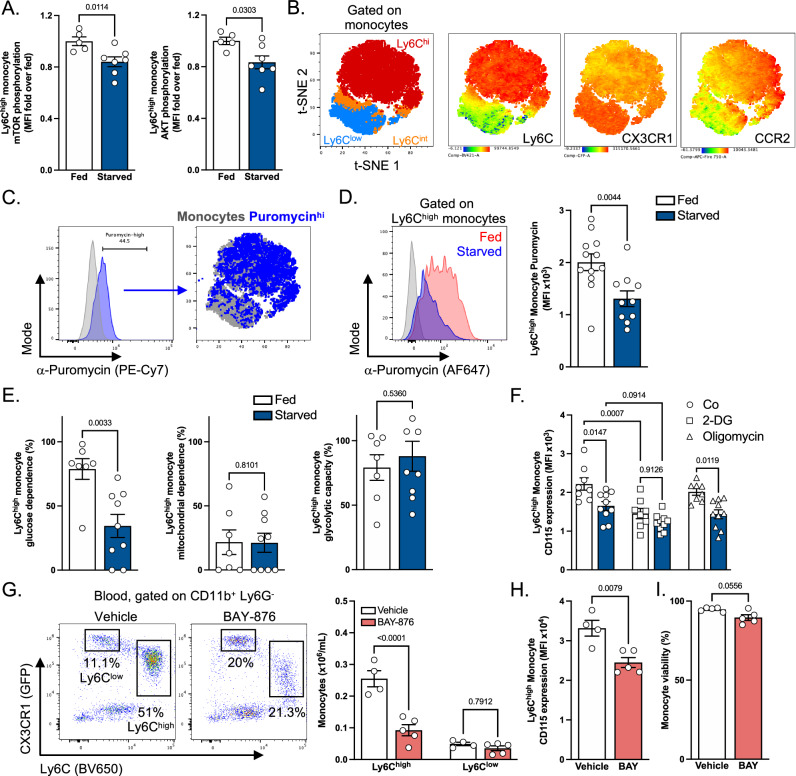
Glucose metabolism is essential for Ly6C^high^ monocyte maintenance. **A** Quantification of mTOR and AKT phosphorylation in Ly6C^high^ monocytes from fed (*n* = 5) and starved (*n* = 7) mice. Data pooled from two independent experiments. **B**, **C** tSNE plots showing **B** identification of blood monocyte subsets and **C** puromycin^+^ cells in C57BL6/J mice at steady state. (**D**) Analysis of puromycin incorporation in monocytes from fed (*n* = 12) or starved (*n* = 10) mice. **E** Analysis of the metabolic profile of Ly6C^high^ monocytes from fed (*n* = 7) or starved (*n* = 9) mice using SCENITH. **F** Quantification of surface CD115 expression in Ly6C^high^ monocytes from fed (*n* = 8) or starved (*n* = 10) mice after ex vivo treatment with vehicle, oligomycin or 2-DG. **D**–**F**: Data pooled from three independent experiments. **G** Quantification of blood Ly6C^high^ and Ly6C^low^ monocytes from CX3CR1^GFP^ mice treated with vehicle (*n* = 4) or 4.5 mg/kg/day BAY-876 (*n* = 5) for 4 days. **H**, **I** Quantification of blood Ly6C^high^ monocyte CD115 MFI (**H**) and viability (**I**) after a 4-day treatment with vehicle (*n* = 4) or 4.5 mg/kg/day BAY-876 (BAY) (*n* = 5) in C57BL6/J mice. Data from one experiment. For statistical analysis, two-sided Mann–Whitney tests were used in (**A**), (**D**), (**E**), (**H**) and (**I**), and two-way ANOVA with Šídák’s multiple comparisons tests were used in (**F**) and (**G**). Data are presented as mean values +/− SEM. See also Fig. S2. Source data are provided as a Source data file.

Next, we sought to determine how starvation impacted intracellular monocyte metabolism. The phosphorylation of AKT and mTOR was reduced on monocytes in starved mice in comparison to fed mice, highlighting their altered metabolic status (Fig. 2A). To analyze modulations in monocyte metabolism, we performed single-cell metabolic profiling using flow cytometry^23^. To apprehend if monocytes were metabolically heterogeneous at steady state, this method was used in conjunction with a panel of antibodies allowing to identify the full blood monocyte differentiation and maturation spectrum (Figs. 2B and S3A). As previously described, Ly6C^low^ monocytes highly expressed CX3CR1, while CCR2 expression was higher on Ly6C^high^ blood monocytes (Fig. 2B). Puromycin incorporation, reflecting protein translation and acting as a surrogate marker of metabolic activity, was found to be higher in Ly6C^high^ monocytes in comparison to Ly6C^low^ monocytes and correlated with CCR2 expression (Fig. 2B, C). On the other hand, the marker of monocyte longevity Treml4 which is highly expressed on Ly6C^low^ monocytes^24^, correlated poorly with CCR2 expression (Figs. 2B and S3A). We then compared the metabolic demands of blood Ly6C^high^ monocytes obtained from fed and starved mice. We observed that Ly6C^high^ monocytes isolated from fed mice incorporated higher puromycin levels when compared to the same monocyte subset in starved animals, indicating a more active metabolic state (Fig. 2D).

Monocyte metabolic activity was previously shown to mainly rely on glucose at steady state^23^. We thus investigated whether Ly6C^high^ monocytes underwent metabolic reprogramming during starvation to adapt to lower glucose and higher NEFA concentrations. We found that Ly6C^high^ monocytes obtained from starved mice had significantly lower glucose dependence in comparison to Ly6C^high^ monocytes in fed mice (Fig. 2E). However, their mitochondrial dependence was similar in both groups, suggesting that Ly6C^high^ monocytes from starved mice reduced their glucose dependence but did not shift their metabolism towards mitochondria-dependent pathways (Fig. 2E). Moreover, monocyte glycolytic capacity remained similar, indicating that Ly6C^high^ monocytes from starved mice were still able to utilize glucose to fuel their metabolism, even though glucose availability was decreased (Fig. 2E). Importantly, CD115 expression on Ly6C^high^ monocytes was decreased by 2-Deoxy-D-Glucose (2-DG) treatment, revealing a key role of glucose metabolism for optimal membrane CD115 expression (Fig. 2F). In contrast, oligomycin had no major effect on CD115 monocyte expression, further suggesting that glucose incorporation, rather than mitochondrial metabolism, is involved in the maintenance of expression of this growth factor receptor (Fig. 2F).

Our data suggest that glucose metabolism positively controls CD115 expression and serum glucose concentration strongly correlates with blood monocyte numbers. To investigate how glucose regulates monocyte homeostasis in vivo, we injected mice with the Glut1-selective pharmacological inhibitor BAY-876 (BAY) (Fig. S3B). Glut1, encoded by *Slc2a1*, is the main glucose transporter in murine myeloid cells^15^. BAY-876 decreased monocyte content in a dose-dependent manner (Fig. S3C). In line with known BAY-876 effects, using 2-NBDG, we observed that glucose entry in Ly6C^high^ monocytes was strongly diminished in BAY-876 treated mice (Fig. S3D). In bone marrow-derived macrophages (BMDMs) stimulated with LPS, BAY-876 treatment inhibited ECAR elevation after glucose administration, suggesting a decrease in glycolysis (Fig. S3E, F). In vivo, BAY-876 administration rapidly and selectively diminished blood Ly6C^high^ monocyte numbers, demonstrating that Glut1-dependent glucose utilization is crucial for their maintenance in physiological conditions (Fig. 2G). Furthermore, BAY-876 triggered a reduction in CD115 expression on Ly6C^high^ blood monocytes, similarly to the effect of starvation and 2-DG (Fig. 2H). We also noticed that BAY-876 administration decreased viability among blood monocytes (Fig. 2I). Taken together these data demonstrated that blood monocytes highly rely on glucose intracellular metabolism for their maintenance and particularly on Glut1-dependent glucose internalization.

### Glut1 inhibition reduces monocyte generation by rewiring myelopoiesis

**Fig. 3:**
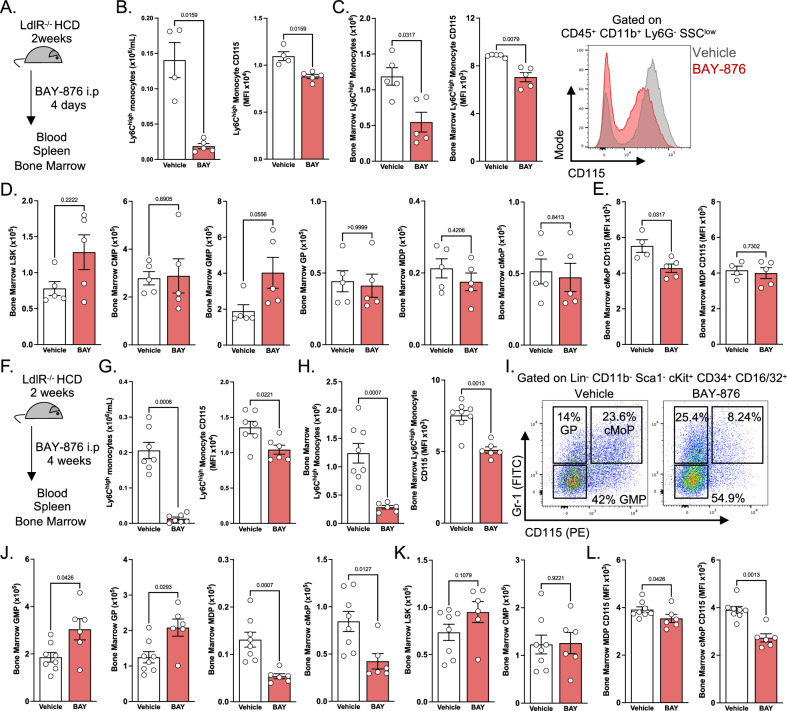
Prolonged BAY-876-mediated Glut1 inhibition promotes hematopoietic skewing towards granulopoiesis. **A** Experimental scheme applying to (**B**), (**C**), (**D**), and (**E**). LdlR^−/−^ mice were fed high cholesterol diet for two weeks before receiving a 4-day treatment with vehicle (*n* = 4) or 4.5 mg/kg/day BAY-876 (BAY) (*n* = 5). These panels represent data from one experiment. **B** Quantification of blood Ly6C^high^ monocyte numbers and CD115 expression after acute vehicle or BAY-876 treatment. **C** Quantification of bone marrow Ly6C^high^ monocyte numbers and CD115 expression after acute vehicle or BAY-876 treatment. **D** Quantification of bone marrow hematopoietic progenitors after acute vehicle or BAY-876 treatment. (**E**) Quantification of surface CD115 expression by bone marrow MDPs and cMoPs after acute vehicle or BAY-876 treatment. **F** Experimental scheme applying to (**G**), (**H**), (**I**), (**J**), and (**K**). LdlR^−/−^ mice were fed high cholesterol diet for two weeks before receiving a 4-week treatment with vehicle (*n* = 7) or 4.5 mg/kg/day BAY-876 (*n* = 6). These panels represent data from one experiment. **G** Quantification of blood Ly6C^high^ monocyte numbers and CD115 expression after chronic vehicle or BAY-876 treatment. **H** Quantification of bone marrow Ly6C^high^ monocyte numbers and CD115 expression after chronic vehicle or BAY-876 treatment. **I** Flow cytometry plots illustrating proportions of granulocyte and monocyte progenitors after chronic BAY-876 treatment. **J**, **K** Quantification of bone marrow hematopoietic progenitors after chronic vehicle or BAY-876 treatment. **L** Quantification of surface CD115 expression by bone marrow MDPs and cMoPs after acute vehicle or BAY-876 treatment. Two-sided Mann–Whitney tests were used for statistical analysis. Data are presented as mean values +/− SEM. See also Fig. S2. Source data are provided as a Source data file.

Monocytosis, high circulating monocyte numbers, is an independent risk factor during atherosclerosis development. To determine the pathophysiological significance of our observations, we decided to investigate whether BAY-876 treatment could impact monocyte behavior and plaque development during atherosclerosis. This disease is characterized by lipid and immune cell, mainly monocyte and macrophage, accumulation inside the vessel intima leading to its narrowing and dysfunction^25,26^. Increased blood monocyte numbers are associated with atherosclerosis progression and their plaque recruitment is crucial to sustain plaque growth in early and advanced disease stages^11,27,28^. To investigate whether Glut1 inhibition modulates monocyte numbers in hypercholesterolemic animals, we administered BAY-876 daily to high cholesterol diet (HCD)-fed atherogenic LdlR^−/−^ mice for 4 days (Fig. 3A). Similarly to our observations in C57BL/6 mice, BAY-876 administration strongly reduced blood Ly6C^high^ monocyte numbers and their membrane CD115 expression in LdlR-deficient animals (Fig. 3B). Of interest, splenic Ly6C^high^ monocyte content was also decreased following BAY-876 injection (Fig. S3G). Their CD115 membrane expression and viability were also reduced in BAY-876 treated mice in comparison to control mice (Fig. S3G,H). Importantly, we noticed that mice injected with BAY-876 presented a strong decrease in bone marrow Ly6C^high^ monocytes (Fig. 3C). These cells displayed lower CD115 surface expression in comparison to vehicle treated mice (Fig. 3C). Because bone marrow monocyte content was affected by BAY-876 administration, we decided to determine the impact of Glut1 pharmacological inhibition on hematopoiesis. Using flow cytometry, we defined several bone marrow progenitor cells and quantified their numbers in vehicle and BAY-876-injected animals (Fig. S3I). Our analyses did not reveal a major modification of LSK, CMP, GP, MDP and cMoP numbers (Fig. 3D). However, GMP numbers tended to be increased following BAY-876 treatment (Fig. 3D). We noticed that BAY-876 reduced CD115 expression on cMoPs, while having no detectable effect on MDPs (Fig. 3E). Taken together, this set of data indicated that the reduced bone marrow, splenic and blood Ly6C^high^ monocyte content was unlikely a consequence of altered myelopoiesis, but rather a cell-autonomous effect of the compound on mature monocytes.

Since atherosclerosis is a chronic inflammatory disease, we decided to investigate whether BAY-876 treatment could keep constantly low Ly6C^high^ monocyte numbers during a long period of time. LdlR-deficient mice were injected daily with BAY-876 or vehicle for 4 weeks (Fig. 3F). In line with our short-term treatment, we detected a very strong decrease in blood Ly6C^high^ monocyte numbers (Fig. 3G). Their CD115 expression was also reduced (Fig. 3G). Bone marrow Ly6C^high^ monocytes were decreased upon BAY-876 treatment and also displayed a lower CD115 expression (Fig. 3H). Analysis of bone marrow myelopoiesis showed that GMPs were increased in the bone marrow of BAY-876 injected mice (Fig. 3I, J). Furthermore, a clear dichotomy appeared at the level of GPs, MDPs and cMoPs. BAY-876 administration led to increased GP numbers and decreased MDPs and cMoPs (Fig. 3I, J). Early progenitors such as LSK cells and CMPs were not affected by Glut1 pharmacologic inhibition (Fig. 3K). These data demonstrated that Glut1 inhibition skewed myelopoiesis towards granulocyte generation at the expense of monocytes (Fig. 3I, J). This was also associated with a reduced CD115 expression on MDPs and cMoPs (Fig. 3L). Thus, upon long-term treatment, preventing Glut1-dependent glucose entry led to decreased monocyte numbers by affecting both myelopoiesis and mature monocyte survival. Importantly, we observed that Glut1 pharmacological inhibition diminished monocyte progenitors while increasing granulocyte generation.

### Glut1 inhibition does not affect atherosclerotic plaque formation and macrophage content despite a major reduction of monocyte numbers

Monocyte recruitment dominates plaque initiation and early growth during atherosclerosis. To test whether BAY-876-induced decrease in blood monocyte content could impact atherosclerosis initiation and early plaque development, we established a 6-week protocol to induce this disease (Fig. S4A). LdlR-deficient mice were fed a HCD for 6 weeks, injected with BAY-876 during the last 4 weeks, and early plaque lesions were analyzed (Fig. S4A). BAY-876-treated mice displayed similar serum cholesterol, glucose, NEFA and TG concentrations (Fig. S4B, C). Surprisingly, plaque area was similar between control and BAY-876-treated animals despite a sustained reduction of blood monocyte numbers (Fig. S4D). These data suggest that compensatory mechanisms are at play and allow for initiation and early plaque growth despite very low numbers of circulating blood monocytes.

**Fig. 4:**
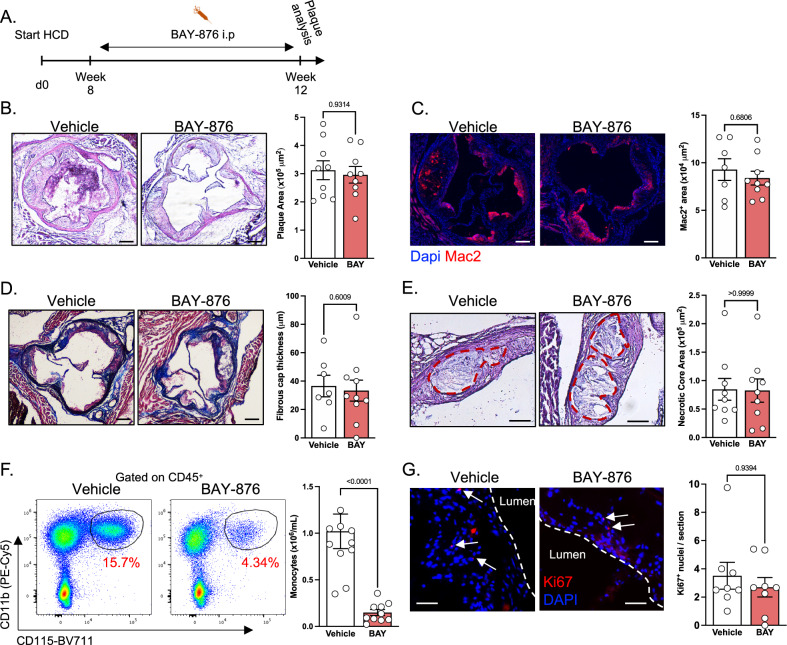
Prolonged BAY-876 treatment does not affect atherosclerosis development. **A** Experimental scheme applying to the entire figure. LdlR^−/−^ mice were fed high cholesterol diet for 8 weeks before receiving vehicle or 4.5 mg/kg/day BAY-876 (BAY) for another 4 weeks. Data pooled from 2 independent experiments. **B** Analysis of plaque area using hematoxylin and eosin staining. Vehicle *n* = 9, BAY-876 *n* = 9. Scale bar: 200 μm. **C** Quantification of plaque macrophage content using Mac2 staining. Vehicle *n* = 7, BAY-876 *n* = 9. Scale bar: 200 μm. **D** Measurement of fibrous cap thickness using Masson’s trichrome staining. Vehicle *n* = 7, BAY-876 *n* = 10. Scale bar: 200 μm. **E** Quantification of necrotic core area using H&E staining. Vehicle *n* = 9, BAY-876 *n* = 9. Scale bar: 100 μm. **F** Quantification of blood monocyte numbers at the end of BAY-876 treatment. Vehicle *n* = 10, BAY-876 *n* = 10. **G** Analysis of intra-plaque proliferation using Ki67 staining. Vehicle *n* = 8, BAY-876 *n* = 8. Scale bar: 50 μm. Two-sided Mann–Whitney tests were used for statistical analysis. Data are presented as mean values +/− SEM. See also Fig. S3. Source data are provided as a Source data file.

To determine the impact of Glut1-dependent glucose metabolism on progression of pre-established plaques, we injected BAY-876 in atherogenic LdlR-deficient mice with already established plaques (8 weeks of high cholesterol diet) (Fig. 4A). Mice injected with BAY-876 had similar body weight as vehicle-treated animals (Fig. S4E). However, the spleen weight of BAY-876-administered mice was dramatically decreased (Fig. S4F). BAY-876 treatment had no major effect on serum glucose concentration (Fig. S4G). This observation was not surprising since the Glut family contains 14 members with distinct cell and tissue expression. Thus, and despite its essential role in glucose uptake in myeloid cells, blocking Glut1 had no substantial effect on systemic glucose levels. Plasma cholesterol concentration also remained similar between the two experimental groups (Fig. S4G).

Histologic analysis showed that plaque area was not modified following BAY-876 administration (Fig. 4B). Plaque macrophage content was not modulated between vehicle and BAY-876-injected mice (Fig. 4C). Fibrous cap thickness measurement demonstrated that BAY-876 administration has no impact on this parameter (Fig. 4D). Finally, the necrotic core size, a direct consequence of defective intraplaque apoptotic cell removal, was not affected by the treatment (Fig. 4E). Using DAPI nuclei staining, we observed that plaque acellular areas were also similar between control and BAY-876-treated mice (Fig. S4H).

Importantly, blood monocyte numbers were 5 times lower in BAY-876-treated mice showing a dramatic and persistent decrease in blood monocyte counts over the 4-week period of treatment (Fig. 4F). This suggested that despite the significant decrease in circulating monocyte numbers, the plaque size and macrophage content were maintained constant by alternative compensatory mechanisms. Thus, we investigated whether BAY-876 triggered increased macrophage proliferation in plaque. Ki67 staining was similar between control and BAY-876 treatment (Fig. 4G) excluding increased local proliferation rates of cells as compensatory mechanism to sustain monocyte and macrophage counts in the atherosclerotic plaques.

### Glut1 inhibition alters monocyte recruitment and differentiation

**Fig. 5:**
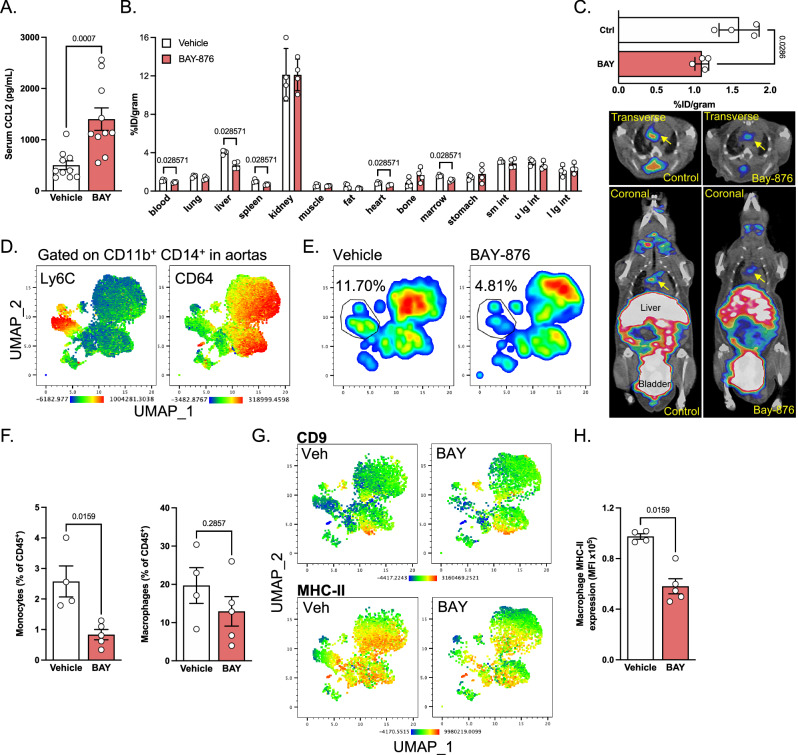
Distribution of monocytes in BAY-876-treated mice. **A** Serum CCL2 levels in LdlR^−/−^ mice fed high cholesterol diet and treated with vehicle (*n* = 10) or 4.5 mg/kg/day BAY-876 (BAY) (*n* = 10) for 4 weeks. Data pooled from two independent experiments. **B** Biodistribution of ^64^Cu-DOTA-ECL1i in LdlR^−/−^ mice fed high cholesterol diet for 2 weeks and treated with vehicle (*n* = 4) or 4.5 mg/kg/day BAY-876 (*n* = 4) for 4 days. Data from one experiment. **C** Quantification of tracer uptake in the aortic arch, as identified with the yellow arrows. Vehicle *n* = 4, BAY-876 *n* = 4. Data from one experiment. **D** Uniform Manifold Approximation and Projection (UMAP) representation of Ly6C and CD64 expression in aortic CD11b^+^ CD14^+^ cells concatenated from LdlR^−/−^ mice treated with vehicle or BAY-876. Data from one experiment. **E** Representative proportions of Ly6C^+^ cells in aortas from LdlR^−/−^ mice treated with vehicle or BAY-876. Data from one experiment. **F** Abundance of monocytes and macrophages in aortas from LdlR^−/−^ mice treated with vehicle (*n* = 4) or BAY-876 (*n* = 5). Data from one experiment. **G** UMAP representation of CD9 and MHC-II expression in CD11b^+^ CD14^+^ aortic cells from LdlR^−/−^ mice treated with vehicle or BAY-876. Data from one experiment. **H** Expression of MHC-II in aortic macrophages from LdlR^−/−^ mice treated with vehicle (*n* = 4) or BAY-876 (*n* = 5). Data from one experiment. Two-sided Mann–Whitney tests were used for statistical analysis. Data are presented as mean values +/− SEM. See also Fig. S4. Source data are provided as a Source data file.

BAY-876 treatment induced increased plasma CCL2 concentration while TNFα level remained unchanged (Figs. 5A and S5A). This suggested that monocyte CCR2-dependent chemotaxis could be specifically modulated by BAY-876 administration, while systemic inflammation, assessed by TNFα concentration, remained equivalent. Thus, we decided to investigate monocyte content in tissues using positron emission tomography and computed tomography (PET/CT) imaging to non-invasively detect CCR2 expression in vivo using a targeted radiotracer (^64^Cu-DOTA-ECL1i)^5,29^. LdlR-deficient mice were fed a HCD and injected with BAY-876 for this purpose. Ex vivo biodistribution studies showed reduced ^64^Cu-DOTA-ECL1i uptake at 1 h post tail vein injection in blood, further confirming the diminished monocyte counts following BAY-876 injection (Fig. 5B). Several tissues including spleen, liver, marrow and heart displayed similar patterns (Fig. 5B). Although BAY-876 treatment did not influence CCR2^+^ cell content in other tissues such as lung, intestine, adipose tissue, kidney and muscle, not a single tissue displayed increased monocyte content after BAY-876 administration (Fig. 5B). Importantly, BAY-876 treatment led to reduced CCR2 tracer uptake in the aortic arches of LdlR^−/−^ mice, where CCR2^+^ monocytes are recruited to promote the development of atherosclerotic plaques (Fig. 5C), suggesting that monocyte recruitment could be affected by Glut1 pharmacological inhibition. We next performed spectral flow cytometry analysis of aortas from HCD-fed LdlR^−/−^ mice after BAY-876 treatment. We focused on CD11b^+^ CD14^+^ myeloid cells in order to characterize monocyte and macrophage content, identified by Ly6C and CD64 expression respectively (Figs. 5D and S5B). Similar to our finding using PET/CT, we observed reduced proportions of Ly6C^+^ monocytes in aortas after BAY-876 treatment (Fig. 5E, F). In contrast, proportion of CD64^+^ macrophages was unaffected by the treatment (Fig. 5F), an observation in agreement with our histological analysis (Fig. 4C). These results suggest that the rate at which monocytes are recruited and differentiate into macrophages might be affected by BAY-876 treatment, ultimately leading to similar plaque macrophage content. We next investigated whether changes in this differentiation pattern could affect the phenotype of plaque macrophages. Monocytes are predicted to differentiate into either CD9^+^ foam cells or MHC-II^+^ inflammatory macrophages after entering plaques^30^. We thus analyzed CD9 and MHC-II expression in CD11b^+^ CD14^+^ aortic cells (Fig. 5G). We identified foam cells as CD11b^+^ CD14^+^ CD64^+^ CD11c^+^ CD9^+^ cells (Fig. S5B), and their abundance in aortas was not affected by BAY-876 treatment (Figs. 5G and S5C). However, MHC-II expression was lower in aortic macrophages from BAY-876-treated mice (Fig. 5G, H). These results suggest that glucose metabolism might regulate monocyte fate after recruitment.

To further characterize how BAY-876 treatment affected monocyte maturation in response to inflammation, we performed a peritonitis assay based on intraperitoneal inoculation of 10 μg zymosan (Z10), which triggers a rapid monocyte recruitment^31^. In order to avoid perturbating the peritoneal space by intraperitoneal injections of BAY-876, we took advantage of the oral bioavailability of this compound^32^ and treated the mice by gavage before inoculating Z10 intraperitoneally (Fig. S5D). We analyzed peritoneal cells the next day and focused on CD11b^+^ CD115^+^ cells that did not express the small peritoneal and large peritoneal macrophage markers CD226 and ICAM2, respectively (Fig. S5E). Numbers of CD226^-^ ICAM2^-^ cells increased in both vehicle and BAY-876-treated animals after Z10 treatment, although these recruited cells were 50% less numerous in BAY-876-treated mice (Fig. S5F). Importantly, we observed a marked difference between this 2-fold reduction in recruited cells compared to the 5-fold reduction we observed in blood Ly6C^high^ monocytes. We next analyzed monocyte maturation via expression of Ly6C and MHC-II^33^, and found that Ly6C^+^ MHC-II^-^ and Ly6C^-^ MHC-II^+^ cells were less numerous in BAY-876-treated mice (Fig. S5F). However, numbers of Ly6C^+^ MHC-II^+^ cells were not affected by the treatment (Fig. S5F). Overall, these results confirm that Glut1 inhibition impacts the differentiation trajectory of recruited monocytes but does not prevent them from giving rise to macrophages.

**Fig. 6:**
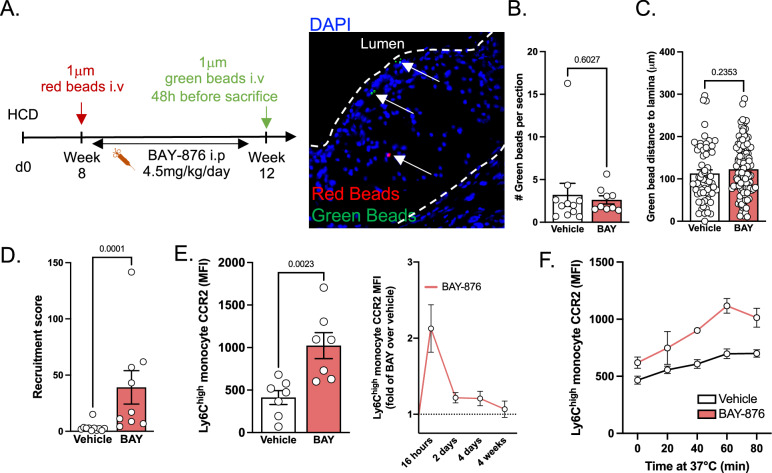
BAY-876 treatment promotes Ly6C^high^ monocyte chemotaxis. **A** Experimental design used to evaluate plaque progression and monocyte recruitment. Image representative of red and green bead presence in plaque. **B** Quantification of green beads in plaque, representing monocyte recruitment. Each dot represents one mouse in which the average number of beads per section was quantified. Vehicle *n* = 10, BAY *n* = 9. **C** Measurement of green bead distance to lamina. Each dot represents a single bead, and beads were observed across several sections and animals. Vehicle *n* = 63 beads, BAY *n* = 103 beads. **D** Recruitment score of monocytes, measured as (number of beads per section)/(number of blood Ly6C^high^ monocytes (×10^6^) per mL). Vehicle *n* = 11, BAY *n* = 9. **E** Analysis of surface CCR2 expression by blood Ly6C^high^ monocytes during vehicle BAY-876 treatment of LdlR^−/−^ mice fed high cholesterol diet. Histogram on the left represents the 16 h timepoint (Vehicle *n* = 7, BAY *n* = 7). Graph on the right is represented as fold change of BAY-876 over mean of vehicle group. Data pooled from 5 independent experiments. 16 h *n* = 10, 2 days *n* = 5, 4 days *n* = 5, 4 weeks *n* = 4. **F** Quantification of surface CCR2 expression on blood Ly6C^high^ monocytes after incubating whole blood from vehicle-treated (*n* = 4) or BAY-treated (*n* = 2) mice at 37 °C for the indicated time to allow recycling of CCR2 to the cell surface. Data from one experiment. Two-sided Mann–Whitney tests were used for statistical analysis. Data are presented as mean values +/− SEM. See also Fig. S4. Source data are provided as a Source data file.

We next sought to functionally test how BAY-876 treatment affects monocyte plaque recruitment. In order to track monocyte recruitment from blood to plaque and their intraplaque distribution, we injected 1 μm fluorescent red beads when we started BAY-876 treatment and 1 μm fluorescent green beads 48 hours before harvesting the aortas and hearts (Fig. 6A)^34,35^. Intraplaque red bead numbers were similar between control and BAY-876-injected mice (Fig. S6A). This observation indicates that similar numbers of blood monocytes accessed the plaque at baseline in both experimental groups. Importantly, the mean distance of red beads to lamina was equal in vehicle and BAY-876-treated animals, indicating that plaque size was similar at the point of inhibitor administration (Fig. S6B). The mean distance between the red beads and the vessel lumen was also comparable in BAY-876 and vehicle injected mice (Fig. S6B). These results support our previous histological observation that plaque area was not modulated by BAY-876 administration (Fig. 4B). Taken together, this set of data demonstrated that plaque growth was similar during the 4 weeks of BAY-876 treatment despite significantly lower circulating monocyte numbers.

Surprisingly, and despite a 5-fold decrease in their circulating monocytes, BAY-876-administered animals had numbers of green beads in plaques similar to control mice, suggesting the recruitment of a comparable number of monocytes (Figs. 6B and S6C). Their distance to the vessel elastic lamina was similar in both experimental groups, further supporting evidence that plaque area was not altered by BAY-876 treatment (Fig. 6C). Since monocyte bead labeling efficiency was similar in vehicle and BAY-876-injected mice, we calculated a recruitment score based on plaque green bead content and blood monocyte numbers (Fig. 6D). BAY-876 treatment strongly reduced blood monocyte numbers while increasing their plaque entry. CCR2 expression, the main chemokine receptor involved in monocyte plaque recruitment, was increased in blood monocytes from BAY-876-injected mice (Fig. 6E). We sought to define the precise kinetics of CCR2 regulation in monocytes upon inhibition of glucose uptake. BAY-876 injection triggered a rapid increase in CCR2 membrane expression at 16 h post-administration (Fig. 6E). Blood monocyte numbers were not statistically different at this early timepoint, although CD115 downregulation was already detectable (Fig. S6D). Monocyte expression of CCR2 then progressively decreased to steady-state levels (Fig. 6E). Importantly, CCR2 recycling was accelerated upon BAY-876 treatment (Fig. 6F). These data indicated that glucose metabolism regulated CCR2 surface expression, at least in part, by modifying its recycling rate. Thus, inhibiting glucose metabolism promoted monocyte recruitment during atherosclerosis progression and increased their CCR2 expression.

### Glut1-dependent glucose uptake regulates CD115 expression through glycosylation in murine and human monocytes

In immune cells, glucose integrates metabolic pathways feeding the pentose phosphate pathway (PPP) and glycolysis. To decipher the relative contribution of myeloid cell glycolysis to monocyte and macrophage functions and the consequences on atherosclerosis development, we generated *Lyz2*^cre^ x *Pfkfb3*^fl/fl^ (PFKFB3^ΔM^) mice. PFKFB3 is the rate-controlling enzyme in glycolysis^36^. We generated bone marrow chimeras by transplanting bone marrow from PFKFB3^ΔM^ or control Lyz2^cre^ mice bearing a Rosa26^LSL-TdTomato^ allele into lethally irradiated atherosclerosis prone LdlR^−/−^ mice. Analysis of their blood immune cell content revealed similar white blood cell counts (WBC), neutrophil and monocyte numbers (Fig. S7A). Furthermore, we observed that monocyte subset numbers were not modified in PFKFB3^ΔM^ mice (Fig. S7A). Importantly, serum cholesterol, LDL cholesterol, and HDL cholesterol levels were similar between both genotypes (Fig. S7B). Glucose and TG concentrations were similar in control and myeloid cell PFKFB3-deficient mice (Fig. S7B). After feeding the mice for 12 weeks on high cholesterol diet, we performed atherosclerotic plaque analysis. Plaque area remained similar in control and PFKFB3^ΔM^ atherogenic mice (Fig. S7C). Moreover, plaque macrophage content (assessed by both Mac2 staining and TdTomato signal) and necrotic core areas were also comparable in both genotypes (Fig. S7D, E). To further assess the impact of PFKFB3-dependent glycolysis on macrophage efferocytosis, we incubated peritoneal macrophages obtained from control and PFKFB3^ΔM^ mice with labeled apoptotic cells. Macrophages were pre-stimulated with either IL-4 (alternative activation) or LPS (classical activation) to rewire their metabolic configuration. In untreated and IL-4 stimulated macrophages, efferocytosis rate was similar in control and PFKFB3-deficient macrophages (Fig. S7F). However, LPS-stimulated PFKFB3^ΔM^ macrophages had improved efferocytosis ability in comparison to control cells activated with LPS (Fig. S7F). Thus, PFKFB3 appeared to reduce efferocytosis selectively in inflammatory macrophages, which are known to activate glycolysis upon LPS stimulation.

**Fig. 7:**
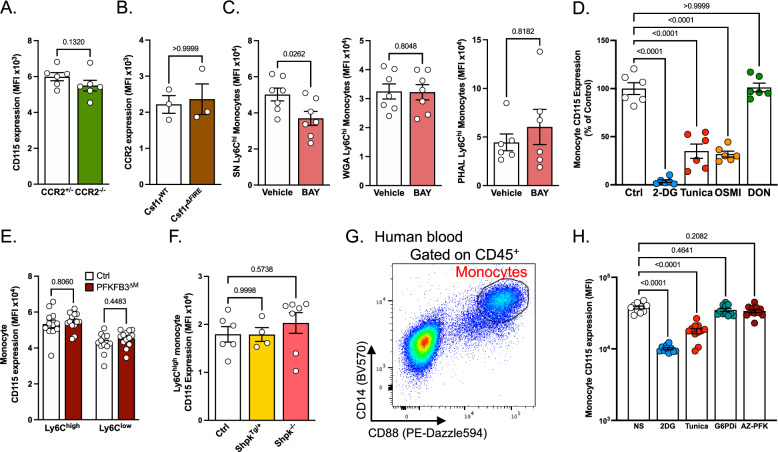
Hexosamine biosynthetic pathway and glycolysis differentially regulate Ly6C^high^ monocyte CD115 and CCR2 expression. **A** Quantification of CD115 expression on blood monocytes from CCR2^gfp/+^ (CCR2^+/−^, *n* = 6) and CCR2^gfp/gfp^ (CCR2^−/−^, *n* = 6) mice. Data pooled from two independent experiments. **B** Quantification of CCR2 expression on blood monocytes from Csf1r^WT^ (*n* = 3) and Csf1r^ΔFIRE^ (*n* = 3) mice. Data representative of three independent experiments. **C** Analysis of surface glycosylation on spleen Ly6C^high^ monocytes incubated overnight with vehicle (SN and WGA *n* = 7, PHAL *n* = 6) or BAY-876 (BAY) (SN and WGA *n* = 7, PHAL *n* = 6). Data pooled from two independent experiments. **D** Quantification of CD115 expression on blood monocytes (from *n* = 6 mice) incubated with vehicle, 2-DG, tunicamycin, OSMI-1 or DON. Data pooled from two independent experiments. **E** Quantification of surface CD115 expression by blood monocytes from control (PFKFB3^fl/fl^, *n* = 12) and PFKFB3^ΔM^ (Lyz2^cre/+^ x PFKFB3^fl/fl^, *n* = 13) mice. Data representative of 3 independent experiments. **F** Quantification of surface CD115 expression by blood Ly6C^high^ monocytes from control (*n* = 6), Shpk^Tg/+^ (*n* = 4) and Shpk^−/−^ (*n* = 7) mice. Data representative of 2 independent experiments. **G** Gating strategy used to identify human blood monocytes after overnight in vitro culture. This strategy applies to Figs. 7H and S8I. **H** Quantification of surface CD115 expression on human blood monocytes (from *n* = 11 healthy donors) treated with 2-DG, tunicamycin, G6PDi or AZ-PFKFB3-67. Two-sided Mann–Whitney tests were used for statistical analysis in (**A**–**C**). One-way ANOVA with Dunnett’s multiple comparison tests were used for statistical analysis in (**D**), (**F**), and (**H**). A two-way ANOVA with Šídák’s multiple comparisons test was used in (**E**). Data are presented as mean values +/− SEM. See also Fig. S6. Source data are provided as a Source data file.

Because blocking Glut1-dependent glucose entry and limiting glucose availability affected both CD115 and CCR2 expression on monocytes, we decided to investigate whether CD115 and CCR2 were both regulated by glucose independently or could be regulating each other. We first analyzed CD115 expression in CCR2^gfp/+^ (CCR2^+/−^) mice^37^ and double knock-in CCR2^gfp/gfp^ mice (CCR2^−/−^) which lack functional CCR2 protein, and did not observe significant difference between the two genotypes (Fig. 7A). We next analyzed Csf1r^ΔFIRE^ mice, which lack the genetic enhancer necessary for expression of CD115 in monocytes and some resident macrophage populations^38^. Importantly, CCR2 expression was not affected by the absence of CD115 (Fig. 7B). Together, these results rule out the hypothesis that CD115 and CCR2 might be directly regulating each other.

We next asked whether a particular metabolic pathway of intracellular glucose utilization could selectively control those molecules. Namely, we aimed to define the contribution of the hexosamine biosynthetic pathway (HBP), glycolysis and PPP to CCR2 and CD115 regulation in monocytes. BAY-876 treatment altered, at least partially, monocyte glycosylation (Fig. 7C). BAY-876 administration reduced Sambucus Nigra (SN) staining on monocytes while having no effect on Wheat germ agglutinin (WGA) and phytohemagglutinin-L (PHAL) (Fig. 7C). These data suggest that preventing glucose entry in monocytes affected preferentially selective types of glycosylation rather than reducing total glycosylation levels. Treatment with tunicamycin, an inhibitor of the hexosamine biosynthetic pathway and glycosylation, decreased membrane CD115 expression on blood monocytes, similar to our observation with 2-DG and BAY-876 (Fig. 7D). This observation suggested that glucose entry in the hexosamine biosynthetic pathway plays a crucial role to sustain optimal monocyte CD115 expression. However, and in contrast to our data with BAY-876 and 2-DG, tunicamycin did not increase surface monocyte CCR2 expression (Fig. S8A). To further explore the role of the hexosamine biosynthetic pathway, we used the O-GlcNAc transferase (OGT) inhibitor OSMI-1. This resulted in CD115 downregulation to a level similar to tunicamycin treatment (Fig. 7D). Inhibiting this pathway tended to promote CCR2 expression, although this did not reach statistical significance (Fig. S8A). Targeting glutaminolysis with the inhibitor DON did not impact CD115 or CCR2 expression, suggesting that glucose-dependent regulations of this receptor occurred independently of glutaminolysis (Figs. 7D and S8A). The analysis of blood monocytes in PFKFB3^ΔM^ mice demonstrated an increased CCR2 expression, similar to our observations with BAY-876 (Fig. S8B). Therefore, glucose flux via PFKFB3 appeared as the regulator of monocyte CCR2 expression (Fig. S8B). In contrast, PFKFB3-deficient monocytes had similar CD115 expression on both Ly6C^high^ and Ly6C^low^ subsets when compared to control mice (Fig. 7E).

Finally, we also investigated PPP contribution to monocyte CD115 and CCR2 expression. To investigate the relative contribution of the PPP to key monocyte and macrophage functions during atherosclerosis we used DHEA, a pharmacological inhibitor targeting glucose-6-phosphate dehydrogenase (G6PD), a rate-limiting enzyme controlling metabolic flux into this pathway (Fig. S8C). G6PD directs glucose-6-phosphate into the PPP. Importantly, DHEA treatment had no major impact on blood monocyte counts (Fig. S8D). Furthermore, monocyte CD115 and CCR2 expression were unaffected following DHEA administration (Fig. S8E). Finally, we analyzed plaque development in LdlR^−/−^ mice treated with the aforementioned inhibitor for 4 weeks. DHEA administration tended to reduce plaque size (Fig. S8F). This observation supports a previous study demonstrating a protective effect of DHEA administration on atherosclerotic plaque development in rabbits^39^. Moreover, DHEA treatment resulted in a reduction of plaque necrosis (Fig. S8F), suggesting that directing glucose flux towards the PPP in macrophages might tone down their efferocytosis capacity. We thus complemented these observations with ex vivo analysis of macrophage efferocytosis. Peritoneal macrophages were treated for 12 h with BAY-876, G6PDi, and DHEA (two pharmacological inhibitors of the initial PPP enzyme G6PD), and cells were then incubated with labeled apoptotic thymocytes. Efferocytosis was not modulated by BAY-876 treatment (Fig. S8G). However, G6PD inhibition by both G6PDi and DHEA led to increased macrophage efferocytosis (Fig. S8G). Taken together, these observations revealed that the PPP plays a key role in the ability of macrophages to uptake and degrade apoptotic cells. We then took advantage of *Shpk* (sedoheptulose kinase) transgenic and deficient mice. Shpk is a key enzyme in the PPP that has been shown to control glucose flux and macrophage activation^18^. CD115 and CCR2 expression on Ly6C^high^ monocytes were similar between control, Shpk-overexpressing (Shpk^Tg/+^) and Shpk-knockout (Shpk^−/−^) mice (Figs. 7D and S8H), suggesting that glucose flux towards the PPP is not the main driver for CD115 and CCR2 regulation (Figs. 7F and S8H). Taken together, our data demonstrated that monocyte CD115 expression is maintained by glucose and the hexosamine biosynthetic pathway, while CCR2 expression shows an independent and inverse regulation by glycolysis.

We next sought to validate our findings using human blood from healthy donors. Whole blood was lysed and cultured overnight in the presence of glucose metabolism inhibitors. Monocytes were then identified by flow cytometry as CD14^+^ CD88^+^ cells (Fig. 7G). 2-DG and tunicamycin treatments both negatively impacted CD115 expression on human monocytes (Fig. 7H), while only 2-DG treatment triggered CCR2 upregulation (Fig. S8I) confirming our murine data. Again, inhibition of the PPP by G6PDi did not significantly impact CD115 nor CCR2 expression (Figs. 7H and S8I). Interestingly, while inhibition of PFKFB3 using the inhibitor AZ-PFKFB3-67 (AZ-PFK) did not impact CD115 expression (Fig. 7H), it triggered a decrease in CCR2 expression (Fig. S8I), a result opposite to our findings in murine PFKFB3-deficient monocytes. These data clearly indicated a conserved regulation of CD115 and CCR2 expression by glucose metabolism in mice and humans, however, the precise mechanism of regulation might slightly differ.

## Discussion

In the present study we defined a key role of glucose on monocyte behavior (Fig. S8J). Glucose metabolism has been shown to control macrophage activation and functions including their ability to engulf and digest apoptotic cells^14,15^. Importantly, genetic Glut1 ablation in myeloid cells (*Lyz2*^cre^ x *Slc2a1*^fl/fl^ mice) triggered a slightly decreased blood monocyte number^16^. How precisely intracellular glucose handling is involved in this process remains to be defined. Of interest, BAY-876 administration diminished even further monocyte numbers in comparison to the aforementioned genetic model. However, despite the strong decrease in blood monocyte numbers, BAY-876-treated mice had similar plaque area as vehicle-injected animals. This observation is in line with data obtained using bone marrow transfer of *Lyz2*^cre^ x *Slc2a1*^fl/fl^ mice into LdlR^−/−^ atherogenic mice^14–16^. During atherosclerosis development, intracellular glucose metabolism supports plaque apoptotic cell removal by macrophages^14,15^. In the present study we demonstrated that preventing Glut1-dependent glucose internalization strongly reduced blood monocyte counts while increasing their CCR2 expression and increasing their plaque recruitment. This compensatory mechanism could compensate for monocyte loss and contribute to plaque growth. Furthermore, our data suggest that monocyte to macrophage differentiation is regulated by glucose flux. Additional studies are required to precisely define whether glucose intracellular metabolism favors or represses the generation of specific monocyte-derived macrophages at steady-state and upon acute or chronic inflammation. Previous study demonstrated that CSF1 triggers increased glucose uptake in bone marrow-derived macrophages^40^, supporting the significance of glucose metabolism to macrophage maturation. Mechanistically, CSF1 induced increased membrane Glut1 expression on macrophages^41^.

How precisely glucose flux is involved in the control of monocyte migration remains to be defined. Upon internalization, glucose could be metabolized into three major pathways, namely glycolysis, the pentose phosphate pathway and hexosamine biosynthetic pathway. Recent studies aimed to define the role of myeloid cell glycolysis in atherosclerosis development. Myeloid cell PFKFB3 haplodeficient ApoE^−/−^ mice displayed a reduced plaque size in comparison to PFKFB3-sufficient control animals^42^. However, myeloid cell selective deletion of PFKFB3 in atherogenic LdlR^−/−^ mice (*Lyz2*^cre^ x *Pfkfb3*^fl/fl^ x *Ldlr*^−/−^ mice), a key enzyme involved in the glycolysis pathway, has no impact on plaque area^43^. The necrotic core area was also similar between *Lyz2*^cre^ x *Pfkfb3*^fl/fl^ x *Ldlr*^−/−^ and control mice^43^. Our data support these observations (Fig. S7), but we also observed an increased monocyte CCR2 expression in PFKFB3-deficient monocytes (Fig. S8B). Therefore, glycolysis might negatively control monocyte migration. How precisely PFKFB3-dependent glucose flux regulates monocyte CCR2 expression and what are the pathophysiological consequences of this regulation require further investigations.

In humans, PFKFB3 expression was associated with unstable plaques^44^. Furthermore, in advanced plaques, PFKFB3 expression colocalized with macrophage CD68 staining and a positive correlation was established between PFKFB3 expression and necrotic core area^44^. These data suggested a potential involvement of PFKFB3-dependent glycolysis to macrophage efferocytosis. While the necrotic core area was similar in PFKFB3^ΔM^ and littermate atherogenic mice (Fig. S7E), we observed that LPS-treated PFKFB3-deficient peritoneal macrophages had increased efferocytosis index in comparison to PFKFB3-sufficient cells (Fig. S7F). These data suggest that PFKFB3-dependent glucose flux could negatively regulate efferocytosis, in particular during macrophage inflammatory responses. A recent study revealed that glycolysis-derived lactate promoted the expression of key efferocytosis receptors (MerTK and LRP1) on macrophages^45^. The authors reported that PFKFB2 played a key role in sustaining macrophage efferocytosis^45^. Thus, PFKFB2 and PFKFB3 could differently contribute to lactate generation and efferocytosis depending on macrophage activation state and the local microenvironment. Our data also revealed that pharmacologic G6PD-inhibiton also increased macrophage efferocytosis (Fig. S8G). Therefore, glucose flux into the PPP is a crucial regulator of macrophage efferocytosis.

Using a starvation approach, we explored associations between nutrient availability and blood monocyte counts. Our data are in agreement with recent findings demonstrating that acute (4 h) and prolonged (20 h) fasting led to decreased blood Ly6C^hi^ monocyte counts^21^. Additionally, it was established that chronic caloric restriction triggered memory T cell accumulation inside the bone marrow compartment at the expenses of blood and peripheral tissues^46^. Importantly, our data indicates that glycemia is the main metabolic parameter regulating monocyte homeostasis and CD115 expression. Our analyses suggested that monocytes are mainly glycolytic and fail to switch towards mitochondrial metabolism upon glucose deprivation and this likely triggers their death and limits their numbers. The precise mechanism why monocytes are poor to adapt their metabolism from glucose to additional substrates utilization remains to be investigated. How starvation affects monocyte Glut1-mediated glucose transport remains to be elucidated. Indeed, glucose transport is regulated by the number of glucose transporters on the cell membrane and by the affinity of the transporters for glucose.

Pharmacologically targeting Glut1 triggered a rapid decrease of CD115 expression on monocytes and their progenitors, while monocyte CCR2 membrane expression was increased. Glut1 inhibition rapidly impacted mature monocytes, while longer treatments also affected myelopoiesis and impaired monocyte generation. Nevertheless, we cannot exclude that BAY-876 treatment might impact other immune or stromal cells. Interestingly, we found that PFKFB3-dependent glycolysis and G6PD-dependent flux into the PPP were not involved in CD115 expression regulation on monocytes (Figs. 7F and S8E). However, pharmacologic inhibition of the hexosamine biosynthetic pathway massively reduced monocyte CD115 expression (Fig. 7D). CD115 contains a heavily glycosylated extracellular ligand-binding domain^47^. How precisely glucose flux through the hexosamine biosynthetic pathway affects CD115 internalization, degradation and cell-surface export remains to be established. Furthermore, to define the N- and O-glycosylation sites involved in CD115 recycling in monocytes could allow to better apprehend the precise function of this receptor.

Lastly, we tested whether our findings were applicable to human biology. Similarly to our observations on murine monocytes, glucose metabolism and in particular the hexosamine biosynthetic pathway, but not glycolysis or the PPP, supported CD115 surface expression. Although 2-DG treatment also promoted CCR2 expression on human monocytes, we found that it was dampened by inhibition of PFKFB3-dependent glycolysis. This highlights glycolysis as an important regulator of CCR2 expression, although the precise mechanisms might differ between species. Together with the role played by PFKFB3 on efferocytosis, our results suggest that PFKFB3-mediated glycolytic flux in myeloid cells might be an important factor in determining plaque evolution and stability. Glucose lowering therapies showed so far limited success in CVD patients^48^. Our data reveal a potential new compensatory mechanism. Indeed, lowering glucose concentration reduced monocyte numbers but increased their CCR2 expression which could facilitate their lesion recruitment. Thus, combining glucose lowering with therapy aiming to finely tune monocyte migration could significantly improve CVD outcome.

### Study limitations

In this study, we report the selective contribution of intracellular glucose metabolism pathways to monocyte homeostasis at steady state and in the context of atherosclerosis development. Our findings rely in part on pharmacological inhibition of Glut1, the main glucose transporter in myeloid cells. The caveat of this approach is Glut1 inhibition on other cell types such as endothelial cells or smooth muscle cells. Glycolysis regulates endothelial cell functions^49^, and modulating their glucose metabolism could potentially affect monocyte chemotaxis and recruitment to plaques. Whether Glut1 inhibition on endothelial cells is responsible for serum CCL2 accumulation remains to be established. Curiously, overexpressing Glut1 on smooth muscle cells increases their CCL2 production which promotes monocyte recruitment and atherosclerosis progression^50^. It appears unlikely that Glut1 inhibition would phenocopy Glut1 overexpression in smooth muscle cells, especially since they express several other glucose transporters^51^. Additional use of pharmacological inhibitors in vitro, in combination with genetic models in vivo, allowed to decipher the role of glucose metabolization through different intracellular metabolic pathways. While our in vitro data indicate that the hexosamine biosynthetic pathway strongly regulates monocyte biology, the relevance of this pathway remains to be addressed in vivo using appropriate genetic models. To our knowledge those genetic models were not yet generated. Indeed, monocytes and macrophages are highly impacted by their local microenvironment which cannot be adequately mimicked using in vitro approaches. Our in vivo genetic targeting of PFKFB3 relies on Lyz2^cre^ expression, which is not restricted to monocytes nor macrophages. Whether PFKFB3 deletion could affect the functions of other cells such as neutrophils remains to be established. Our observations could be complemented by use of additional models restricted to monocytes or neutrophils (Ly6G^cre^ mice).

## Methods

The present research complies with all relevant ethical regulations. Human participants provided written informed consent through the Centre Hospitalier Universitaire de Nice. All animal procedures were approved by local ethical committees as listed below.

### Experimental models

#### Mouse models

Wild-type C57BL/6J (Jax #000664), CX3CR1^gfp^ (B6.Cg-Ptprca Cx3cr1^tm1Litt/LittJ^, Jax # #008451), Lyz2^cre^ (B6.129P2-*Lyz2*^*tm1(cre)Ifo*^/J, Jax #004781), R26^TdTomato^ (B6.Cg-Gt(ROSA)26Sor^tm9(CAG-tdTomato)Hze^/J, Jax #007909), and LdlR^−/−^ (B6.129S7-Ldlrtm1Her/J, Jax #002207) mice used in this study were maintained under C57BL/6J background and originally purchased from Janvier Labs. PFKFB3^flox^ mice^49^ were kindly provided by Dr. Peter Carmeliet and crossed to Lyz2^cre^ mice. *Shpk* mouse strains were in a C57BL/6N background and heterozygote Shpk overexpressing transgenic mice (Shpk^Tg/+^) were developed in the Haschemi Lab. For this purpose, mouse Shpk mRNA coding sequence (NM_029031.3) was cloned into pCAGGS plasmid^52^ (GenBank: LT727518.1, kindly provided by the BCCM/LMBP Plasmid collection) and sequence integrity was verified by DNA sequencing. The transgene (insert) was generated using Sal I and HIND III restriction sites with a total size of 3.7 kB, including regulatory elements. Transgenic animals were successfully generated with the help of Thomas Rülicke (University of Veterinary Medicine Vienna) and Biomodels Austria by pronuclear transgene microinjection using fertilized mouse embryos of the C57BL/6N background. The *Shpk*^*−/−*^ mouse strain (RRID: MMRRC_043666-UCD) was obtained from the MMRRC at University of California at Davis and was donated by Kent Lloyd, D.V.M., University of California, Davis. CCR2^GFP^ mice^37^ (B6(C)-Ccr2tm1.1Cln/J, Jax #027619) were provided by Dr. Marco Colonna. Csf1r^ΔFIRE^ mice^38^ were provided by Dr. David Hume to Dr. Marc Bajénoff, and both researchers kindly agreed to share the mice with our group. Experimental and control animals were co-housed, and littermate controls were used as often as possible. Animals of mixed sex and similar age (7–12 weeks old) were used within each cohort. Since we did not observe any sex-specific phenotypes, we decided to group data from male and female mice together in experiments where mice of both sexes were used. All mice were bred and housed in specific pathogen-free conditions maintained in facilities in the Mediterranean Center of Molecular Medicine (INSERM U1065, Université Côte d’Azur), Washington University in Saint Louis animal facility, the University of Minnesota Medical School Research Animal Resources facility, the Medical University of Vienna animal facility or the Centre d’Immunologie de Marseille Luminy facility. An ambient temperature of ~20–23 °C was maintained, with a 12/12-h light/dark cycle and food available ad libitum. Animals were euthanized by cervical dislocation. Animal protocols required for experimentation other than organ collection were authorized by the French Ministry of Higher Education and Research upon approval of the local ethical committee (CIEPAL Azur) at Université Côte d’Azur, and by the Institutional Animal Care and Use Committee (IACUC) at Washington University in Saint Louis and University of Minnesota Medical School.

#### Human samples

Veinous blood collection was supervised and performed by the Centre Hospitalier Universitaire de Nice on healthy donors who provided informed consent. Samples consist of peripheral blood from 6 women and 5 men, all aged 22 to 30 years old. Data from males and females were grouped since no sex-specific phenotypes were observed.

### Genotyping

DNA was extracted from ear biopsies by incubation with 50 mM NaOH for 30 min at 95 °C. DNA was amplified by PCR using DreamTaq Green PCR Master Mix (2X) (Thermo Scientific) and primers indicated in Supplementary Table 1. PCR products were visualized on a 2% agarose gel.

### Atherosclerosis induction

To promote atherosclerosis progression, LdlR^−/−^ mice were fed high cholesterol diet (TD88137, Sniff) for the duration indicated on figures or in figure legends. For bone marrow transplant experiments, LdlR^−/−^ mice were irradiated with 7 Gy of X-rays, transplanted the next day with freshly extracted bone marrow and allowed to rest for 4 weeks before starting western diet.

### In vivo treatments

#### Administration of pharmacological inhibitors

BAY-876 (MedChemExpress #HY-100017) and DHEA (Sigma #252805) were solubilized in DMSO and further diluted in 0,5% Methylcellulose for intraperitoneal injections. Mice in control groups received an equivalent dose of DMSO diluted in 0.5% Methylcellulose. Doses are indicated in the corresponding figure legends.

#### Monocyte tracking assay

LdlR^−/−^ mice on high cholesterol diet were injected with red or green (Fluoresbrite® YG Microspheres, polysciences #17154) 1 μm fluorescent beads (diluted ¼ in PBS) at timepoints indicated in figure legends. Bead presence in plaques was assessed by fluorescence microscopy. Monocyte recruitment score was calculated as (number of beads per section)/(number of blood Ly6C^high^ monocytes).

#### Zymosan-induced peritonitis

C57BL/6J mice were administered vehicle or BAY-876 by oral gavage (4.5 mg/kg/day) for 4 days before receiving an intraperitoneal injection of 10 μg zymosan solubilized in 100 μL PBS and analyzed the next day.

### Flow cytometry

Tissues were harvested after cervical dislocation and washed in PBS. Splenocytes were prepared by gently crushing the spleen on a 70 μm strainer in flow buffer (PBS containing 1% BSA and 2 mM EDTA). Peritoneal cells were obtained by performing lavage with 5 mL flow buffer. Aortas were washed and carefully dissected to remove surrounding adipose tissue, before being digested using 100 μg/mL DNAse I and 300 μg/mL Liberase^TM^ TL (Roche). Bone marrow cells were prepared by flushing femurs and tibias with flow buffer. Blood was drawn from the submandibular vein and collected in heparinized tubes or Eppendorf tubes containing 15 μl 500 mM EDTA. Leukocytes were counted using a veterinary hematology analyzer (Exigo H400). Red blood cells were lysed from all single cell suspensions using BD Pharm Lyse lysing solution (BdBiosciences cat #555899). Cells were washed with PBS, stained with violet Live/Dead fixable viability dye (Thermofisher cat #L34955), washed in flow buffer and then stained. For intracellular staining, cells were fixed and permeabilized using Miltenyi Foxp3 staining buffer (cat #130-093-142). For CCR2 staining in Fig. 6I, blood was collected in heparin tubes and then incubated at 37 °C for the indicated time before staining. All antibodies were used 1/200. A list of all antibodies used is provided in Supplementary Table 2. Flow cytometry data were acquired using a BD FACS Canto II and a Cytek Aurora cytometer with 5 laser configuration. All analyses, including unsupervised t-SNE analysis, were performed using FlowJo software (Tree Star).

### Metabolite analyses

#### Colorimetric quantification of metabolites

Glycerol, TG, and NEFA contents were measured from serum. Free glycerol reagent and standard were used according to the manufacturer’s protocol. NEFA-HR2 R1 + R2 FUJIFILM were used according to the manufacturer’s protocol. Glucose, cholesterol, carnitin, and ketone body levels were measured using a Mindray analyzer.

### Seahorse

For Seahorse metabolic analysis, 1 × 10^5^ bone marrow cells from wild-type mice were plated in a Seahorse Bioscience culture plate and cultured in RPMI medium containing 10% FBS, 2 mM L-Glutamine, 50 U/mL Penicillin, 50 μg/mL Streptomycin and 50 ng/mL recombinant murine M-CSF. Medium was changed after 3 days. On the 7th day of culture, medium was refreshed (without M-CSF) and cells received a 1-h pre-treatment with BAY-876 (50 ng/mL) or vehicle before being stimulated (or not) by addition of 100 ng/mL LPS. Six hours post LPS stimulation, medium was replaced with Seahorse assay medium, cells were incubated at 37 °C in the absence of CO_2_ for 45 min and then placed in the XF96 Seahorse analyzer. OCR and ECAR were measured using the glycolysis stress test procedure consisting of successive treatment with glucose (10 mM), oligomycin (1 μM) and finally 2-DG (50 mM). Biological replicates were used in each experiment.

### SCENITH analysis

The method was performed as described in ref. ^23^. SCENITH reagents kit (inhibitors, puromycin, and antibodies) were obtained from www.scenith.com/try-it and used according to the provided protocol for ex vivo analysis of myeloid cells. Blood was collected in heparinized tubes by submandibular bleeding. Whole blood was incubated at 37 °C for 30 min with Puromycin (10 µg/mL) and either Control, 2-Deoxy-D-Glucose (100 mM), Oligomycin (1 µM), 2-Deoxy-D-Glucose + Oligomycin or Harringtonine (2 µg/mL). Red blood cells were then lyzed using cold BD Pharm Lyse buffer. Cells were first stained with Live/Dead fixable viability dye, washed, and incubated with Fc Block (2.4G2, BioXcell) before surface staining. Cells were fixed and permeabilized using Foxp3 fixation/permeabilization buffer (Miltenyi) and then stained with anti-Puromycin (Alexa Fluor 647). Cells that were not incubated with Puromycin were used as a negative control for anti-Puromycin signal. Cells that received surface staining but no anti-Puromycin staining (full minus one) were used to measure subset-specific autofluorescence in the AF647 channel, and this background signal was subtracted. Glucose dependence, Mitochondrial dependence and Glycolytic capacity were calculated as previously described^23^.

### Ex vivo glucose uptake assay

Blood was collected in heparinized tubes by submandibular bleeding. Whole blood was incubated at 37 °C for 15 min with 2-NBDG (Thermofisher cat# N13195) and then directly put on ice. 2-NBDG uptake was assessed by flow cytometry. Cells that were not incubated with 2-NBDG served as a negative control.

### Ex vivo lectin assay

Wild-type mice were treated with vehicle of BAY-876 intraperitoneally. Twenty-four hours later, whole blood was drawn from the submandibular vein, red blood cells were lysed and cells were stained at 37 °C for 15 min with 10 mg/mL biotinylated lectins in RPMI medium containing 1 mg/ml BSA. Lectins were from Vector labs. After washing, cells were incubated with PE-conjugated streptavidin for 15 min together with anti-CD11b, CD115, and Ly6C and analyzed by flow cytometry.

### In vitro cell treatments

Spleens or blood from CX3CR1^GFP^ mice were harvested and gently crushed in a 70 μm sieve to isolate cells. Red blood cells were lysed and 1 × 10^6^ splenocytes were used for the experiment. For analysis of human monocytes, veinous blood was drawn from healthy human volunteers and peripheral blood mononuclear cells were isolated using Ficoll-Paque^TM^ PLUS (Cytiva). Cells were treated overnight in RPMI medium (containing 10% FBS, 2 mM L-Glutamine, 50 U/mL Penicillin, 50 μg/mL Streptomycin) containing vehicle, Tunicamycin (0.2 μg/mL), 2-DG (10 mM), DON (1 μM) or OSMI-I (25 μM), G6PDi (0.5 μM) or AZ-PFKFB3-67 (0.1 μM). For efferocytosis experiments, 1 × 10^5^ peritoneal cells were left to adhere for 6 h, washed and treated overnight with vehicle, LPS (100 ng/mL), IL-4 (20 ng/mL), G6PDi (0.5 μM) or DHEA (1 μM).

### Efferocytosis

Thymii from C57BL/6J mice were harvested and mechanically dissociated. Cells were filtered on 70μm nylon filters (Falcon), pelleted and resuspended in RPMI medium supplemented with 10% FBS. Apoptosis was induced by UV exposure at 312 nm for 10 min. The cells were maintained in culture for additional 2 h. Apoptotic cells were labeled with CellTrace™ Violet Cell Proliferation kit (ThermoFisher) according to the manufacturer’s instructions. Labeled apoptotic cells were washed twice with PBS before use. The stained apoptotic cells were added at a 5:1 ratio on plated macrophages for 30 min. Cells were washed 3 times and macrophages were stained and analyzed for apoptotic cell content by flow cytometry.

### ELISA assays

Blood was collected by submandibular bleeding to prepare serum. Serum was kept at −20 °C until further analysis. CCL2 and TNFα levels were measured using R&D kits (cat # DY479 and DY410, respectively).

### Histology and immunostaining

Mouse hearts were perfused through the left ventricle with PBS, harvested and fixed overnight in 4% paraformaldehyde and 30% sucrose solution. Hearts were then washed with PBS and embedded in OCT. Sections of 10 µm were cut using the cryostat CM350 and mounted on Superfrost Plus slides (Thermo Scientific). Sections were preserved at −20 °C until further analysis by hematoxylin and eosin staining, Masson’s trichrome stain or immunofluorescence using DAPI and Mac2 (Cedarlane cat# CL8942AP, used 1/5000) detected with Cy3-conjugated secondary antibody (Jacksonimmuno cat# 212-165-104).

### Positron emission tomographic imaging/computed tomography (PET/CT) and post-PET biodistribution of ^64^Cu-DOTA-ECL1i

For PET/CT, 45 to 60 min dynamic scan was performed after injection of ^64^Cu-DOTA-ECL1i (3.7 MBq in 100 μL saline) via tail vein with Inveon PET/CT system (Siemens, Malvern, PA). The PET images were reconstructed with the maximum a posteriori algorithm and analyzed by Inveon Research Workplace. The organ uptake was calculated as percent injected dose per gram of tissue in 3-dimensional regions of interest without the correction for partial volume effect. Right after PET/CT, the mice were euthanized by cervical dislocation. Organs of interest were collected, weighed, and counted in a Beckman 8000 gamma counter (Beckman, Fullerton, CA). Standards were prepared and measured along with the samples to calculate the percentage of the injected dose per gram of tissue.

### Statistical analysis

All data are represented in mean ± SEM. Statistical analysis was performed with GraphPad Prism 10 as indicated in each figure legend. Presence of statistically significant differences between conditions are indicated as follows: ns *p* > 0.05; *p < 0.05; **p < 0.01; ***p < 0.001; ****p < 0.0001.

### Reporting summary

Further information on research design is available in the Nature Portfolio Reporting Summary linked to this article.

## Supplementary information

Supplementary Information

Reporting Summary

## Source data

Source Data

Transparent Peer Review file

## Data Availability

No new transcriptomic, proteomic or metabolomic datasets were generated during the study. All data supporting the findings of the study are presented in the main manuscript or in the supplementary information files. Requests relative to the flow cytometry and imaging data presented herein should be directed to corresponding authors (Stoyan.Ivanov@univ-cotedazur.fr or Alexandre.Gallerand@univ-cotedazur.fr) due to still-ongoing analysis for further exploitation of the data. Access to our data shall be granted permanently to researchers who provide written research aims and are affiliated with a recognized institution. We aim to answer and provide access to requested data within 30 days. Source data are provided with this paper.

## References

[1] Jakubzick, C. V., Randolph, G. J. & Henson, P. M. Monocyte differentiation and antigen-presenting functions. *Nat. Rev. Immunol.* **17**, 349–362 (2017). 28436425 10.1038/nri.2017.28

[2] Serbina, N. V., Jia, T., Hohl, T. M. & Pamer, E. G. Monocyte-mediated defense against microbial pathogens. *Annu. Rev. Immunol.* **26**, 421–452 (2008). 18303997 10.1146/annurev.immunol.26.021607.090326 PMC2921669

[3] Ingersoll, M. A. et al. Comparison of gene expression profiles between human and mouse monocyte subsets. *Blood* **115**, e10–19 (2010). 19965649 10.1182/blood-2009-07-235028 PMC2810986

[4] Serbina, N. V. & Pamer, E. G. Monocyte emigration from bone marrow during bacterial infection requires signals mediated by chemokine receptor CCR2. *Nat. Immunol.* **7**, 311–317 (2006). 16462739 10.1038/ni1309

[5] Gallerand, A. et al. Brown adipose tissue monocytes support tissue expansion. *Nat. Commun.* **12**, 5255 (2021). 34489438 10.1038/s41467-021-25616-1 PMC8421389

[6] Dolfi, B. et al. Unravelling the sex-specific diversity and functions of adrenal gland macrophages. *Cell Rep.* **39**, 110949 (2022). 35705045 10.1016/j.celrep.2022.110949 PMC9210345

[7] Carlin, L. M. et al. Nr4a1-dependent Ly6C(low) monocytes monitor endothelial cells and orchestrate their disposal. *Cell* **153**, 362–375 (2013). 23582326 10.1016/j.cell.2013.03.010 PMC3898614

[8] Auffray, C. et al. Monitoring of blood vessels and tissues by a population of monocytes with patrolling behavior. *Science* **317**, 666–670 (2007). 17673663 10.1126/science.1142883

[9] Chapman, C. M., Beilby, J. P., McQuillan, B. M., Thompson, P. L. & Hung, J. Monocyte count, but not C-reactive protein or interleukin-6, is an independent risk marker for subclinical carotid atherosclerosis. *Stroke* **35**, 1619–1624 (2004). 15155967 10.1161/01.STR.0000130857.19423.ad

[10] Johnsen, S. H. et al. Monocyte count is a predictor of novel plaque formation: a 7-year follow-up study of 2610 persons without carotid plaque at baseline the Tromso Study. *Stroke* **36**, 715–719 (2005). 15746459 10.1161/01.STR.0000158909.07634.83

[11] Tacke, F. et al. Monocyte subsets differentially employ CCR2, CCR5, and CX3CR1 to accumulate within atherosclerotic plaques. *J. Clin. Invest*. **117**, 185–194 (2007). 17200718 10.1172/JCI28549 PMC1716202

[12] Qiao, J. H. et al. Role of macrophage colony-stimulating factor in atherosclerosis: studies of osteopetrotic mice. *Am. J. Pathol.* **150**, 1687–1699 (1997). 9137093 PMC1858194

[13] Caputa, G., Castoldi, A. & Pearce, E. J. Metabolic adaptations of tissue-resident immune cells. *Nat. Immunol.* **20**, 793–801 (2019). 31213715 10.1038/s41590-019-0407-0

[14] Morioka, S. et al. Efferocytosis induces a novel SLC program to promote glucose uptake and lactate release. *Nature* **563**, 714–718 (2018). 30464343 10.1038/s41586-018-0735-5 PMC6331005

[15] Freemerman, A. J. et al. Myeloid Slc2a1-deficient murine model revealed macrophage activation and metabolic phenotype are fueled by GLUT1. *J. Immunol.* **202**, 1265–1286 (2019). 30659108 10.4049/jimmunol.1800002 PMC6360258

[16] Flynn, M. C. et al. Transient intermittent hyperglycemia accelerates atherosclerosis by promoting myelopoiesis. *Circ. Res* **127**, 877–892 (2020). 32564710 10.1161/CIRCRESAHA.120.316653 PMC7486277

[17] Wang, Y. T. et al. Metabolic adaptation supports enhanced macrophage efferocytosis in limited-oxygen environments. *Cell Metab*. 10.1016/j.cmet.2022.12.005 (2022). 10.1016/j.cmet.2022.12.005 PMC9908853 36584675

[18] Haschemi, A. et al. The sedoheptulose kinase CARKL directs macrophage polarization through control of glucose metabolism. *Cell Metab.* **15**, 813–826 (2012). 22682222 10.1016/j.cmet.2012.04.023 PMC3370649

[19] Yurdagul, A. Jr. et al. Macrophage metabolism of apoptotic cell-derived arginine promotes continual efferocytosis and resolution of injury. *Cell Metab.* **31**, 518–533.e510 (2020). 32004476 10.1016/j.cmet.2020.01.001 PMC7173557

[20] Ampomah, P. B. et al. Macrophages use apoptotic cell-derived methionine and DNMT3A during efferocytosis to promote tissue resolution. *Nat. Metab.*10.1038/s42255-022-00551-7 (2022). 10.1038/s42255-022-00551-7 PMC9050866 35361955

[21] Jordan, S. et al. Dietary intake regulates the circulating inflammatory monocyte pool. *Cell* **178**, 1102–1114.e1117 (2019). 31442403 10.1016/j.cell.2019.07.050 PMC7357241

[22] Janssen, H. et al. Monocytes re-enter the bone marrow during fasting and alter the host response to infection. *Immunity* **56**, 783–796.e787 (2023). 36827982 10.1016/j.immuni.2023.01.024 PMC10101885

[23] Arguello, R. J. et al. SCENITH: a flow cytometry-based method to functionally profile energy metabolism with single-cell resolution. *Cell Metab.* **32**, 1063–1075.e1067 (2020). 33264598 10.1016/j.cmet.2020.11.007 PMC8407169

[24] Briseno, C. G. et al. Distinct transcriptional programs control cross-priming in classical and monocyte-derived dendritic cells. *Cell Rep.* **15**, 2462–2474 (2016). 27264183 10.1016/j.celrep.2016.05.025 PMC4941620

[25] Back, M., Yurdagul, A. Jr., Tabas, I., Oorni, K. & Kovanen, P. T. Inflammation and its resolution in atherosclerosis: mediators and therapeutic opportunities. *Nat. Rev. Cardiol.* **16**, 389–406 (2019). 30846875 10.1038/s41569-019-0169-2 PMC6727648

[26] Randolph, G. J. Mechanisms that regulate macrophage burden in atherosclerosis. *Circ. Res.* **114**, 1757–1771 (2014). 24855200 10.1161/CIRCRESAHA.114.301174 PMC4059102

[27] Swirski, F. K. et al. Ly-6Chi monocytes dominate hypercholesterolemia-associated monocytosis and give rise to macrophages in atheromata. *J. Clin. Invest*. **117**, 195–205 (2007). 17200719 10.1172/JCI29950 PMC1716211

[28] Rogacev, K. S. et al. CD14++CD16+ monocytes independently predict cardiovascular events: a cohort study of 951 patients referred for elective coronary angiography. *J. Am. Coll. Cardiol.* **60**, 1512–1520 (2012). 22999728 10.1016/j.jacc.2012.07.019

[29] Williams, J. W. et al. Thermoneutrality but not UCP1 deficiency suppresses monocyte mobilization into blood. *Circ. Res.* **121**, 662–676 (2017). 28696252 10.1161/CIRCRESAHA.117.311519 PMC5718914

[30] Patterson, M. T. et al. Trem2 promotes foamy macrophage lipid uptake and survival in atherosclerosis. *Nat. Cardiovasc. Res.* **2**, 1015–1031 (2023). 38646596 10.1038/s44161-023-00354-3 PMC11031198

[31] Louwe, P. A. et al. Recruited macrophages that colonize the post-inflammatory peritoneal niche convert into functionally divergent resident cells. *Nat. Commun.* **12**, 1770 (2021). 33741914 10.1038/s41467-021-21778-0 PMC7979918

[32] Siebeneicher, H. et al. Identification and optimization of the first highly selective GLUT1 inhibitor BAY-876. *ChemMedChem* **11**, 2261–2271 (2016). 27552707 10.1002/cmdc.201600276 PMC5095872

[33] Jakubzick, C. et al. Minimal differentiation of classical monocytes as they survey steady-state tissues and transport antigen to lymph nodes. *Immunity* **39**, 599–610 (2013). 24012416 10.1016/j.immuni.2013.08.007 PMC3820017

[34] Potteaux, S. et al. Suppressed monocyte recruitment drives macrophage removal from atherosclerotic plaques of Apoe -/- mice during disease regression. *J. Clin. Invest.* **121**, 2025–2036 (2011). 21505265 10.1172/JCI43802 PMC3083793

[35] Williams, J. W. et al. Limited macrophage positional dynamics in progressing or regressing murine atherosclerotic plaques-brief report. *Arterioscler Thromb. Vasc. Biol.* **38**, 1702–1710 (2018). 29903736 10.1161/ATVBAHA.118.311319 PMC6202234

[36] Tanner, L. B. et al. Four key steps control glycolytic flux in mammalian cells. *Cell Syst.* **7**, 49–62.e48 (2018). 29960885 10.1016/j.cels.2018.06.003 PMC6062487

[37] Satpathy, A. T. et al. Notch2-dependent classical dendritic cells orchestrate intestinal immunity to attaching-and-effacing bacterial pathogens. *Nat. Immunol.* **14**, 937–948 (2013). 23913046 10.1038/ni.2679 PMC3788683

[38] Rojo, R. et al. Deletion of a Csf1r enhancer selectively impacts CSF1R expression and development of tissue macrophage populations. *Nat. Commun.* **10**, 3215 (2019). 31324781 10.1038/s41467-019-11053-8 PMC6642117

[39] Gordon, G. B., Bush, D. E. & Weisman, H. F. Reduction of atherosclerosis by administration of dehydroepiandrosterone. A study in the hypercholesterolemic New Zealand white rabbit with aortic intimal injury. *J. Clin. Invest* **82**, 712–720 (1988). 2969922 10.1172/JCI113652 PMC303568

[40] Hamilton, J. A., Vairo, G. & Lingelbach, S. R. CSF-1 stimulates glucose uptake in murine bone marrow-derived macrophages. *Biochem. Biophys. Res. Commun.* **138**, 445–454 (1986). 3488736 10.1016/0006-291x(86)90301-3

[41] Chang, M. et al. Phosphatidylinostitol-3 kinase and phospholipase C enhance CSF-1-dependent macrophage survival by controlling glucose uptake. *Cell Signal* **21**, 1361–1369 (2009). 19376223 10.1016/j.cellsig.2009.04.003

[42] Guo, S. et al. Gene-dosage effect of Pfkfb3 on monocyte/macrophage biology in atherosclerosis. *Br. J. Pharm.* **179**, 4974–4991 (2022). 10.1111/bph.15926 PMC10420406 35834356

[43] Tillie, R. et al. Partial inhibition of the 6-phosphofructo-2-kinase/fructose-2,6-bisphosphatase-3 (PFKFB3) enzyme in myeloid cells does not affect atherosclerosis. *Front Cell Dev. Biol.* **9**, 695684 (2021). 34458258 10.3389/fcell.2021.695684 PMC8387953

[44] Poels, K. et al. Inhibition of PFKFB3 hampers the progression of atherosclerosis and promotes plaque stability. *Front Cell Dev. Biol.* **8**, 581641 (2020). 33282864 10.3389/fcell.2020.581641 PMC7688893

[45] Schilperoort, M., Ngai, D., Katerelos, M., Power, D. A. & Tabas, I. PFKFB2-mediated glycolysis promotes lactate-driven continual efferocytosis by macrophages. *Nat. Metab.*10.1038/s42255-023-00736-8 (2023). 10.1038/s42255-023-00736-8 PMC10050103 36797420

[46] Collins, N. et al. The bone marrow protects and optimizes immunological memory during dietary restriction. *Cell* **178**, 1088–1101.e1015 (2019). 31442402 10.1016/j.cell.2019.07.049 PMC6818271

[47] Pixley, F. J. & Stanley, E. R. CSF-1 regulation of the wandering macrophage: complexity in action. *Trends Cell Biol.* **14**, 628–638 (2004). 15519852 10.1016/j.tcb.2004.09.016

[48] Eckel, R. H., Bornfeldt, K. E. & Goldberg, I. J. Cardiovascular disease in diabetes, beyond glucose. *Cell Metab.* **33**, 1519–1545 (2021). 34289375 10.1016/j.cmet.2021.07.001 PMC8411849

[49] De Bock, K. et al. Role of PFKFB3-driven glycolysis in vessel sprouting. *Cell* **154**, 651–663 (2013). 23911327 10.1016/j.cell.2013.06.037

[50] Wall, V. Z. et al. Smooth muscle glucose metabolism promotes monocyte recruitment and atherosclerosis in a mouse model of metabolic syndrome. *JCI Insight*10.1172/jci.insight.96544 (2018). 10.1172/jci.insight.96544 PMC6124428 29875324

[51] Pyla, R., Poulose, N., Jun, J. Y. & Segar, L. Expression of conventional and novel glucose transporters, GLUT1, -9, -10, and -12, in vascular smooth muscle cells. *Am. J. Physiol. Cell Physiol.* **304**, C574–589 (2013). 23302780 10.1152/ajpcell.00275.2012 PMC3671567

[52] Niwa, H., Yamamura, K. & Miyazaki, J. Efficient selection for high-expression transfectants with a novel eukaryotic vector. *Gene* **108**, 193–199 (1991). 1660837 10.1016/0378-1119(91)90434-d

