# Supplementary material for: Glucose metabolism controls monocyte homeostasis and migration but has no impact on atherosclerosis development in mice
Source: Nat Commun. 2024 Oct 19;15:9027. doi: 10.1038/s41467-024-53267-5 (PMC11489573; doi:10.1038/s41467-024-53267-5)
Supplement: Supplementary file 1 — Supplementary Information [file 41467_2024_53267_MOESM1_ESM.pdf]

Supplementary information for :

Glucose metabolism controls monocyte homeostasis and migration but has no impact on atherosclerosis development in mice.

Authors:

Alexandre Gallerand[1,2#], Bastien Dolfi[1,2], Marion I. Stunault[2], Zakariya Caillot[1], Alexia Castiglione[1,2], Axelle Strazzulla[1], Chuqiao Chen[3], Gyu Seong Heo[4], Hannah Luehmann[4], Flora Batoul[2], Nathalie Vaillant[2], Adélie Dumont[2], Thomas Pilot[5], Johanna Merlin[2], Fairouz N. Zair[1], Jerome Gilleron[2], Adeline Bertola[1], Peter Carmeliet[6], Jesse W. Williams[7], Rafael J. Arguello[8], David Masson[5], David Dombrowicz[9], Laurent Yvan-Charvet[2], Denis Doyen[1,10], Arvand Haschemi[3], Yongjian Liu[4], Rodolphe R. Guinamard[1,2] and Stoyan Ivanov[1,2#]

Affiliations:

[1] Université Côte d'Azur, CNRS, LP2M, Nice, France.

[2] Université Côte d'Azur, INSERM, C3M, Nice, France.

[3] Department of Laboratory Medicine, Medical University of Vienna, 1090, Vienna, Austria.

[4] Department of Radiology, Washington University School of Medicine, Saint Louis, MO, USA.

[5] Université Bourgogne Franche-Comté, LNC UMR1231, F-21000 Dijon, France.

[6] Laboratory of Angiogenesis and Vascular Metabolism, Center for Cancer Biology (CCB), VIB, Department of Oncology, Leuven Cancer Institute (LKI), KU Leuven, Leuven, 3000, Belgium.

[7] Center for Immunology, Department of Integrative Biology and Physiology, University of Minnesota Medical School, Minneapolis, MN, USA.

[8] Aix Marseille University, CNRS, INSERM, CIML, Centre d'Immunologie de Marseille-Luminy, Marseille, France.

[9] Univ.Lille, INSERM, CHU Lille, Institut Pasteur de Lille, U1011-EGID, 59000 Lille, France.

[10] Médecine Intensive Réanimation, Hôpital Pasteur, CHU de Nice, Nice, France

[#] Corresponding authors

Correspondence to:

Stoyan.IVANOV@univ-cotedazur.fr

Alexandre.gallerand@univ-cotedazur.fr

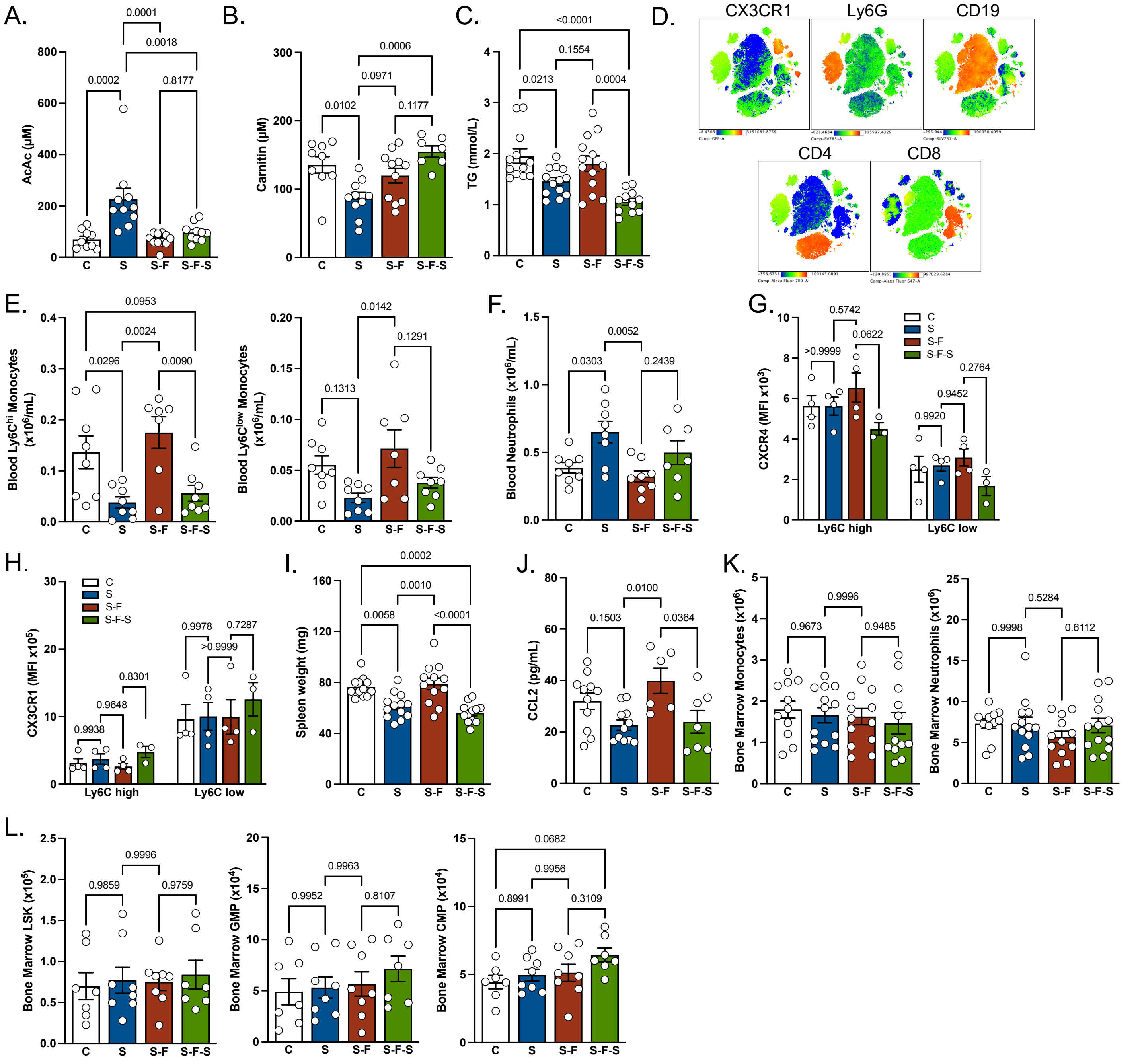

Supplementary figure 1. Impact of starvation on monocytes and neutrophils.
(**A, B, C**) Quantification of serum acetoacetic acid (C n=9, S n=10, S-F n=11, S-F-S n=10) (A), carnitin (C n=9, S n=10, S-F n=11, S-F-S n=7) (B) and triglyceride (C n=12, S n=13, S-F n=13, S-F-S n=11) levels. (**D**) t-SNE plots representing expression of the markers used to identify cell types in Figure 1E, according to the gating strategy in Figure S2A. (**E, F**) Quantification of blood monocyte subset (C n=8, S n=8, S-F n=7, S-F-S n=8) and neutrophil (C n=8, S n=8, S-F n=8, S-F-S n=7) numbers using flow cytometry. (**G, H**) Expression of CXCR4 (G) and CX3CR1 (H) by blood Ly6C^{high} and Ly6C^{low} monocytes. (C n=4, S n=4, S-F n=4, S-F-S n=3). (**I**) Variations of spleen weight following starvation-refeeding challenge. (C n=12, S n=13, S-F n=12, S-F-S n=12). (**J**) Measurement of serum CCL2 levels by ELISA. (C n=11, S n=11, S-F n=6, S-F-S n=7). (**K**) Quantification of bone marrow monocyte (C n=11, S n=13, S-F n=13, S-F-S n=12) and neutrophil (C n=11, S n=13, S-F n=12, S-F-S n=13) numbers. (**L**) Quantification of bone marrow LSK (C n=7, S n=13, S-F n=13, S-F-S n=12), GMP (C n=7, S n=8, S-F n=8, S-F-S n=7) and CMP (C n=7, S n=8, S-F n=8, S-F-S n=7) following starvation-refeeding challenge. Data pooled from 3 independent experiments. One-way ANOVA with Tukey's multiple comparison tests were used in panels A-F and I-L. A two-way ANOVA with Tukey's multiple comparison tests were used in panels G and H. Data are presented as mean values +/- SEM. Related to figure 1. Source data are provided as a Source Data file.

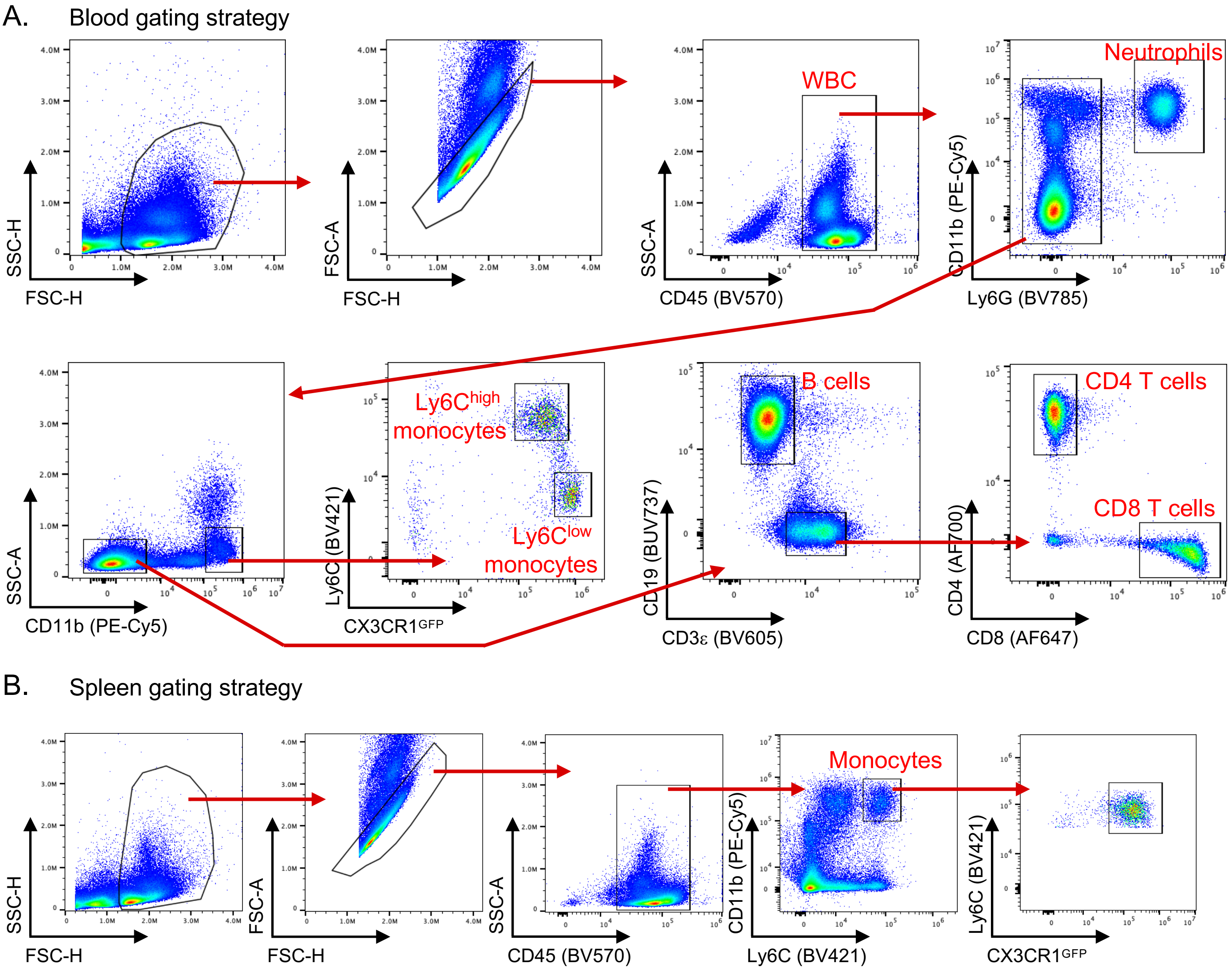

Supplementary figure 2. Blood and spleen gating strategies.
(**A**) Gating strategy used to identify blood white blood cell subsets. (**B**) Gating strategy used to identify monocytes in spleen. This figure relates to all panels showing analysis of blood or spleen monocytes throughout the paper.

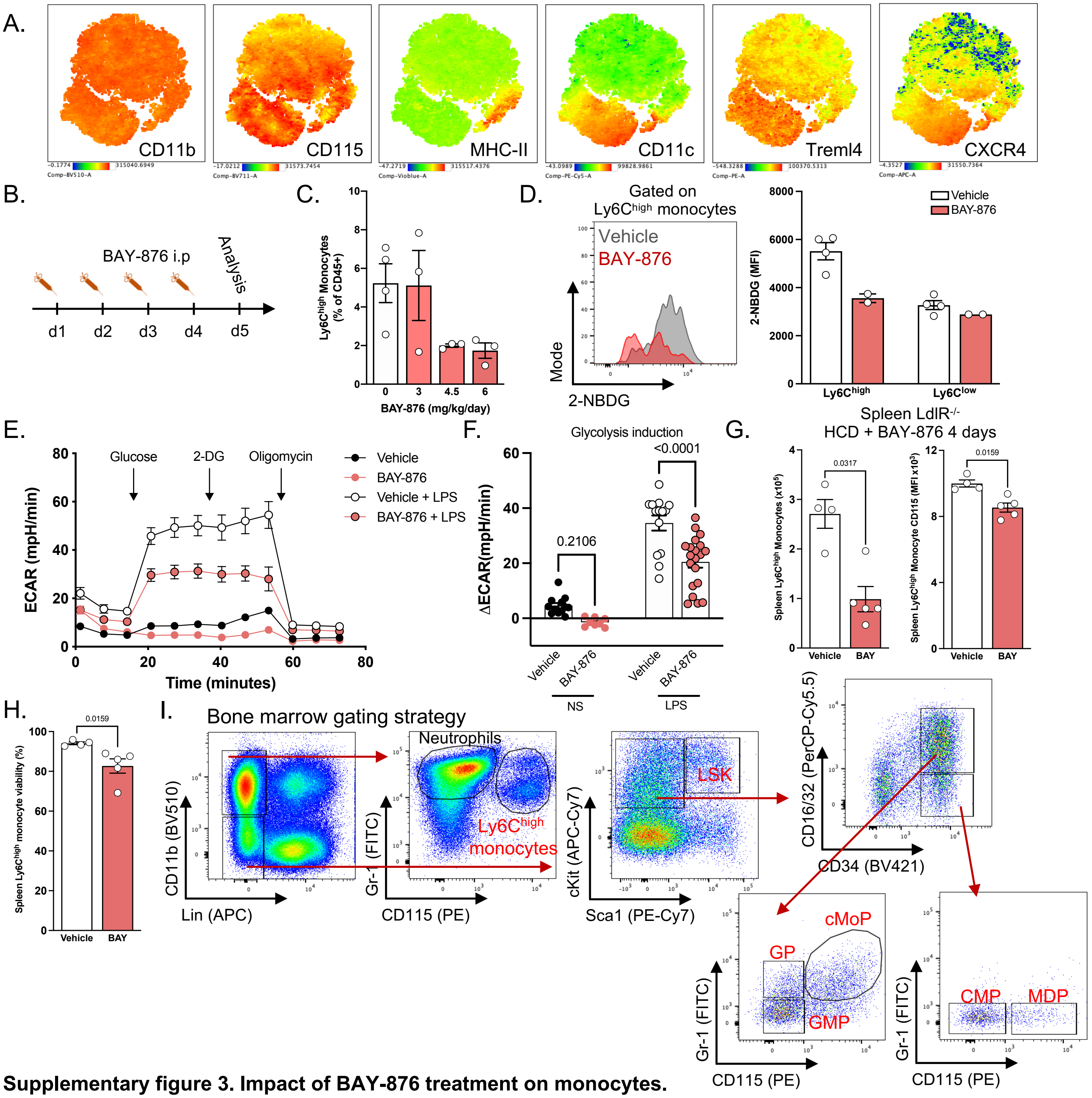

Supplementary figure 3. Impact of BAY-876 treatment on monocytes.

(**A**) tSNE plots showing expression of markers used to identify monocyte subsets in figure 2B. (**B**) Experimental scheme used to analyze impact of short-term BAY-876 treatment. (**C**) Proportions of Ly6C^{high} monocytes among CD45^{+} cells after a 4-day treatment with vehicle (n=4) or increasing doses of BAY-876 (n=3 per dose). Data from one experiment. (**D**) Representative histogram (left) and quantification (right) of 2-NBDG signal in monocytes after a 4-day treatment with vehicle (n=4) or 4,5mg/kg/day BAY-876 (n=2). Data from one experiment. (**E, F**) Seahorse analysis of bone marrow-derived macrophages treated with vehicle (n=11) or BAY-876 (n=8) and stimulated with LPS (vehicle n=14, BAY n=20), using the glycolysis stress test protocol. Data represented as raw ECAR measurement (E) or glycolytic capacity (F). Data pooled from three independent experiments using biological replicates. (**G, H**) Quantification of spleen Ly6C^{high} numbers (G), CD115 MFI (G) and viability (H) in LdlR$^{-/-}$ mice following a 4-day treatment with vehicle (n=4) or BAY-876 (n=5). Data representative of at least three independent experiments. (**I**) Gating strategy used to identify bone marrow monocytes, neutrophils and HSPCs. Lineage (Lin) includes anti-B220, anti-Ter119, anti-NK1.1, anti-CD3. This gating strategy applies to all panels showing analysis of bone marrow cells throughout the paper. A two-way ANOVA with Bonferroni's multiple comparison test was used in panel F. Two-sided Mann-Whitney tests were used for statistical analysis in panels G and H. Data are presented as mean values +/- SEM. Related to figure 2. Source data are provided as a Source Data file.

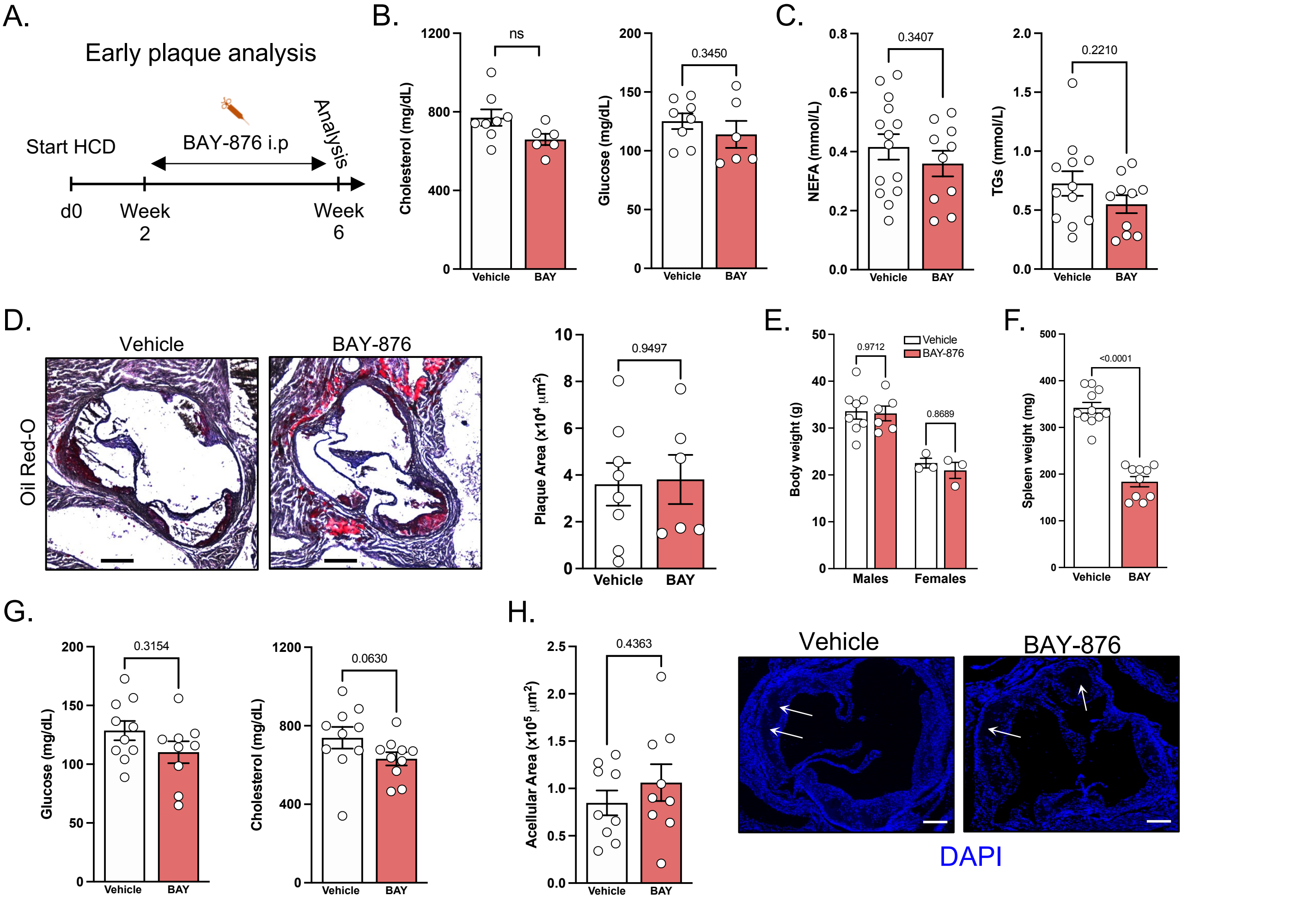

Supplementary figure 4. Impact of BAY-876 treatment on atherosclerosis development.
(**A**) Experimental design used for panels B-H to test impact of early BAY-876 treatment on plaque initiation. (**B-C**) Serum levels of cholesterol and glucose (B) (vehicle n=8, BAY n=6), NEFA and TG (C) (vehicle n=14 and 12 respectively, BAY n=10) in $LdlR^{-/-}$ mice fed high cholesterol diet for two weeks and then treated with BAY-876 for 4 weeks. (**D**) Representative images and quantification of early plaque area in vehicle (n=8) and BAY-876-treated (n=6) $LdlR^{-/-}$ mice, using oil-red-O staining. (**E, F**) Body (E) and spleen (F) weight from $LdlR^{-/-}$ mice fed high cholesterol diet for 8 weeks and then treated with vehicle (n=8 males and 3 females) or BAY-876 (n=6 males and 3 females) for 4 weeks. (**G**) Serum glucose (vehicle n= 10, BAY n=9) and cholesterol (vehicle n=10, BAY n=10) levels from $LdlR^{-/-}$ mice fed western diet for 8 weeks and then treated with vehicle or BAY-876 for 4 weeks. (**H**) Analysis of plaque acellular area, identified by DAPI staining, from $LdlR^{-/-}$ mice fed high cholesterol diet for 8 weeks and then treated with vehicle (n= 9) or BAY-876 (n=9) for 4 weeks. Panels B, D, E and F represent data from one experiment. Panel C represents pooled data from two experiments. Panels G-H represent data pooled from two independent experiments. A two-way ANOVA with Šídák's multiple comparisons test was used in panel E. Two-sided Mann-Whitney tests were used in panels B-D and F-H for statistical analysis. Data are presented as mean values +/- SEM. Related to figure 3. Source data are provided as a Source Data file.

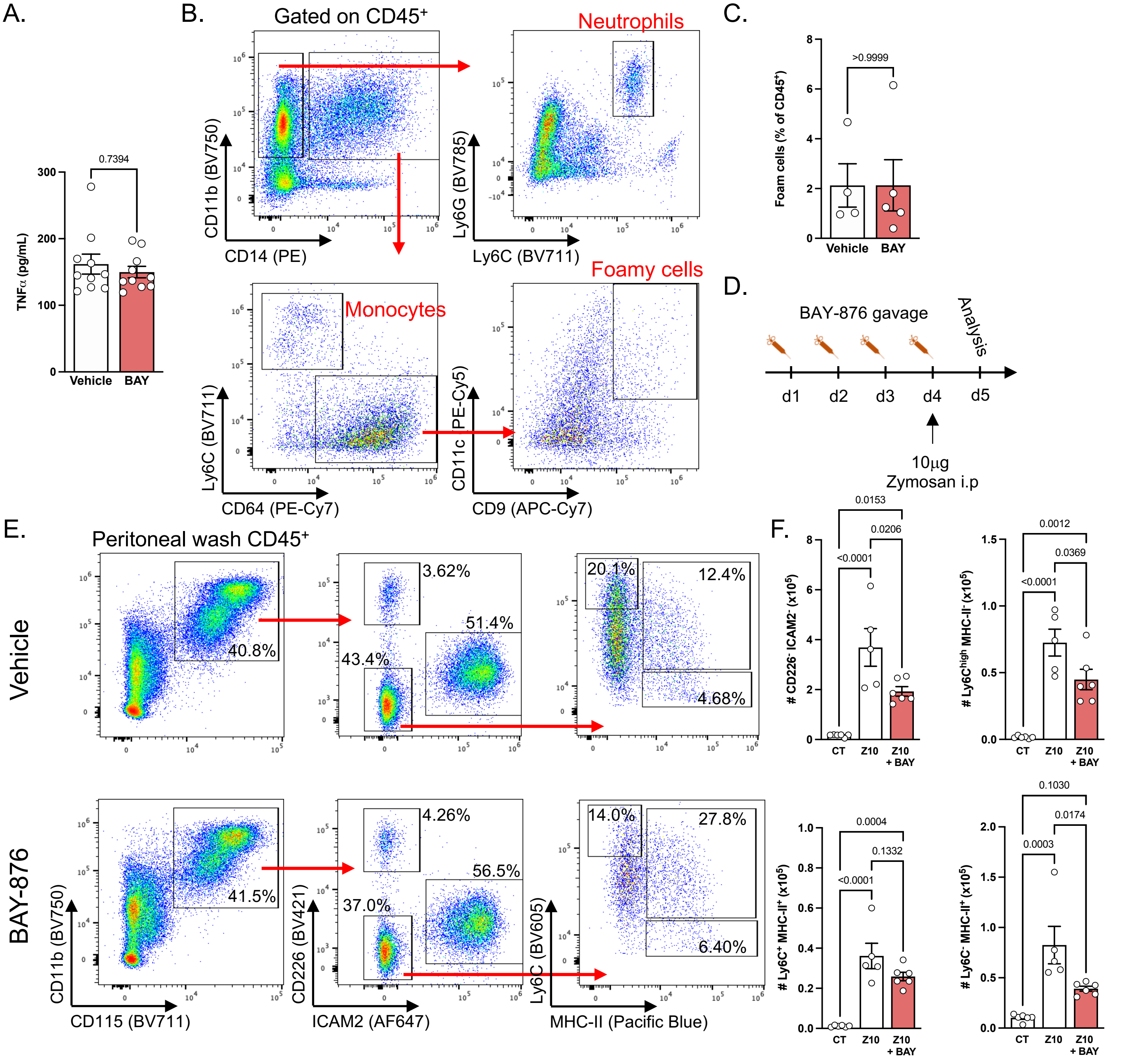

Supplementary figure 5. Impact of BAY-876 treatment on tissue monocytes and macrophages.

(**A**) Serum TNFa levels in LdlR$^{-/-}$ mice treated with vehicle (n=10) or BAY-876 (n=10). (**B**) Gating strategy used for analysis of aortic monocytes and macrophages. (**C**) Proportions of foamy macrophages in aortas from LdlR$^{-/-}$ mice treated with vehicle (n=4) or BAY-876 (n=5). Data from one experiment. (**D**) Experiment scheme used to analyze monocyte recruitment to the peritoneal cavity after zymosan inoculation. (**E**) Gating strategy used to analyze monocyte-derived cells in peritoneal cavity. (**F**) Quantification of monocyte-derived cells in peritoneal cavity at steady state (n=6) or after zymosan challenge in vehicle-treated (n=5) and BAY-876-treated (n=6) mice. Data from one experiment. One-way ANOVA with Tukey's multiple comparisons test were used for statistical analysis in panel F. Two-sided Mann-Whitney tests were used for statistical analysis in panels A and C for statistical analysis. Data are presented as mean values +/- SEM. Related to figure 5. Source data are provided as a Source Data file.

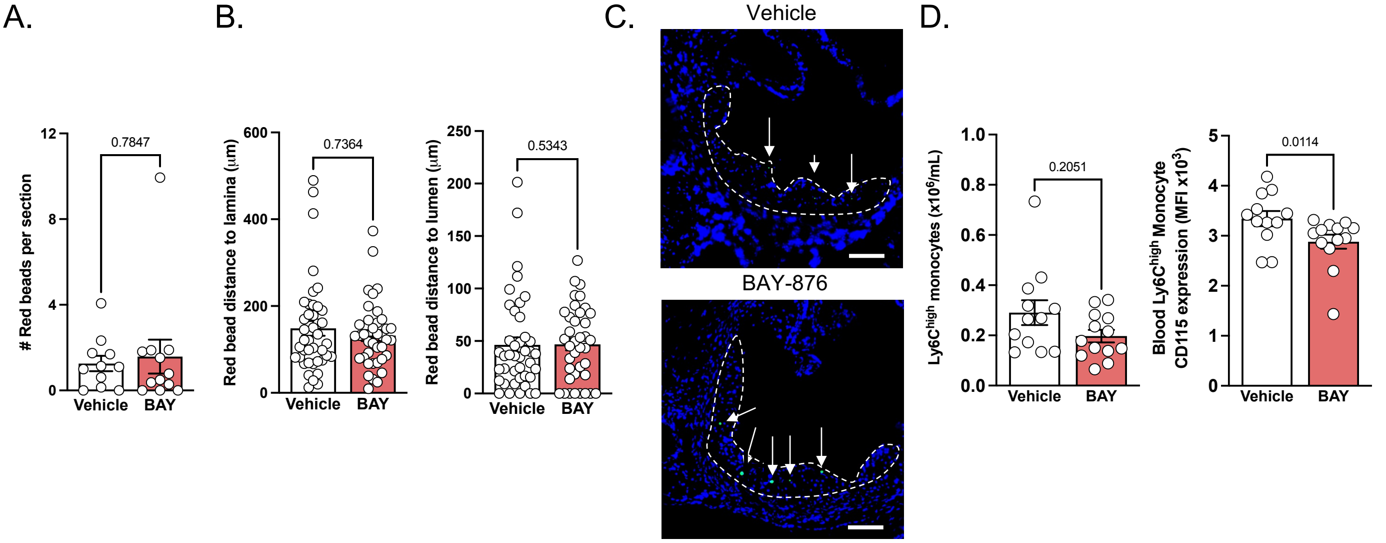

Supplementary figure 6. Impact of BAY-876 treatment on monocyte recruitment.
(**A, B**) Analysis of red bead numbers (vehicle n=11, BAY n=12) (**A**) and distance to lamina and lumen (vehicle n=41 beads, BAY n=38 beads) (**B**) in plaque from LdlR$^{-/-}$ mice fed western diet for 8 weeks and treated with vehicle or BAY-876 (4,5mg/kg/day) for the following 4 weeks. (**C**) Images representative of green bead presence in plaque from vehicle and BAY-876-treated LdlR$^{-/-}$ mice. (**D**) Quantification of blood Ly6C^{high} numbers and CD115 expression 16hours after the first administration of vehicle (n=12) or BAY-876 (n=13). Data pooled from 2 independent experiments. Two-sided Mann-Whitney tests were used for statistical analysis. Data are presented as mean values +/- SEM. Related to figure 6. Source data are provided as a Source Data file.

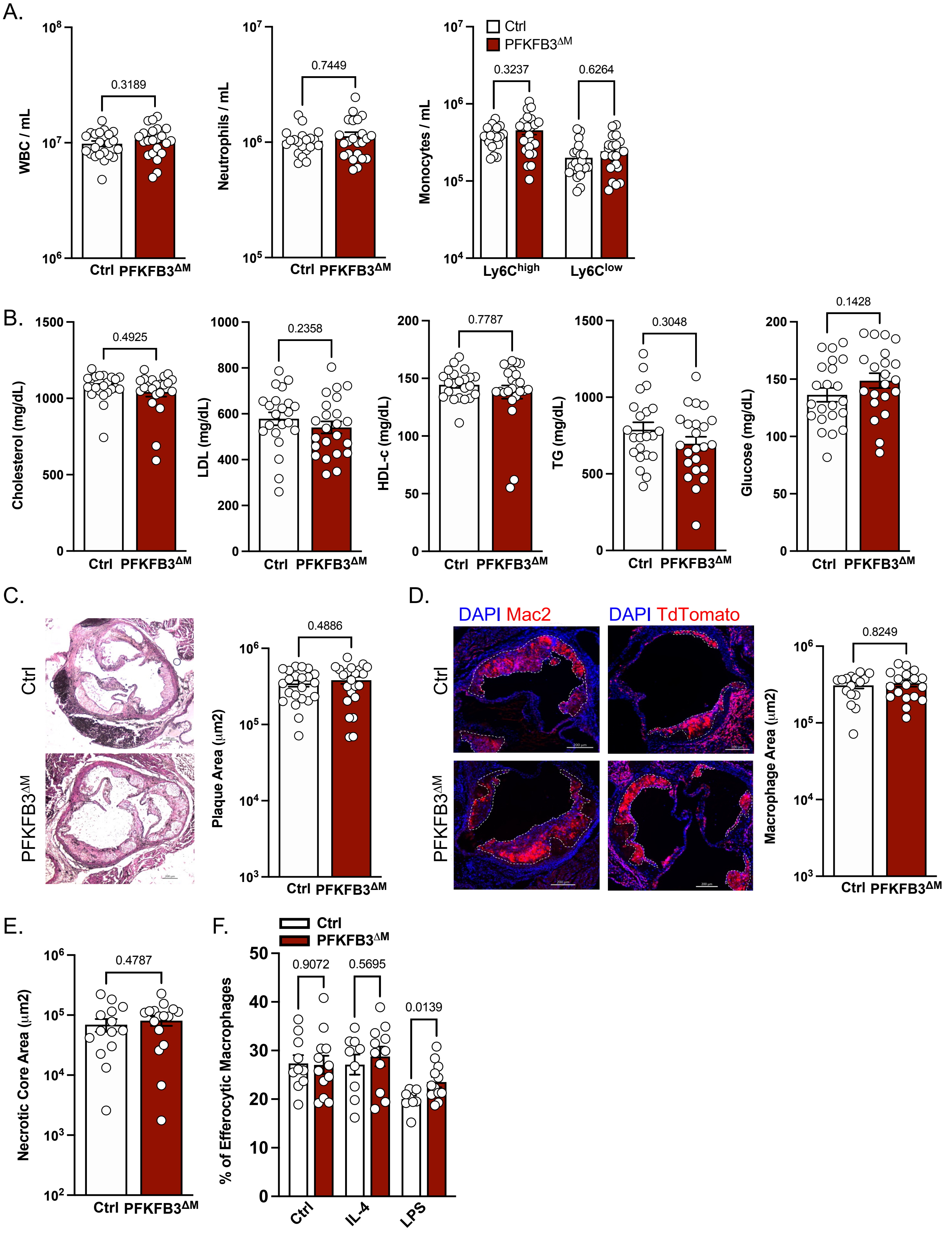

A.
WBC / mL
0.3189
Ctrl PFKFB3ΔM
Neutrophils / mL
0.7449
Monocytes / mL
Ctrl
PFKFB3ΔM
0.3237
0.6264
Ly6Chigh
Ly6Clow
B.
Cholesterol (mg/dL)
0.4925
LDL (mg/dL)
0.2358
HDL-c (mg/dL)
0.7787
TG (mg/dL)
0.3048
Glucose (mg/dL)
0.1428
C.
Ctrl
PFKFB3ΔM
Plaque Area (μm2)
0.4886
D.
DAPI Mac2
DAPI TdTomato
Macrophage Area (μm2)
0.8249
E.
Necrotic Core Area (μm2)
0.4787
F.
% of Efferocytic Macrophages
0.9072
0.5695
0.0139
Ctrl
IL-4
LPS

Supplementary figure 7. Role of Pfkfb3-mediated myeloid cell glycolysis in atherosclerosis.
Bone marrow from Lyz2$^{cre/+}$ Pfkfb3$^{flox/flox}$ R26^{TdTomato} (PFKFB3^{DM}) or Pfkfb3$^{flox/flox}$ R26^{TdTomato} (Ctrl) mice was transplanted into lethally irradiated LdlR$^{-/-}$ mice. Recipients were rested for 4 weeks and then fed western diet for 12 weeks. Data pooled from three independent experiments. (**A**) Quantification of blood total leukocytes (n=22 per genotype), neutrophils (ctrl n=20, PFKFB3^{DM} n=23), Ly6C^{high} (ctrl n=21, PFKFB3^{DM} n=23) and Ly6C^{low} monocytes (ctrl n=22, PFKFB3^{DM} n=23) in control and PFKFB3^{DM} bone marrow chimeras. (**B**) Serum metabolic profile of control (n=22) and PFKFB3^{DM} (n=23) bone marrow chimeras. (**C**) Quantification of plaque area in control (n=22) and PFKFB3^{DM} (n=22) bone marrow chimeras. (**D**) Analysis of plaque macrophage content, represented by Mac2 staining and presence of TdTomato reporter, in control (n=16) and PFKFB3^{DM} (n=18) bone marrow chimeras. (**E**) Quantification of plaque necrotic area in control (n=16) and PFKFB3^{DM} (n=18) bone marrow chimeras. (**F**) Analysis of efferocytic capacity of large peritoneal macrophage from control and PFKFB3^{dM} mice stimulated with vehicle (n=10 and n=12), IL-4 (n=9 and n=11) or LPS (n=9 and n=12). Two-way ANOVA with Šídák's multiple comparisons tests were used for statistical analysis in panels A (third panel) and F. Two-sided Mann-Whitney tests were used for statistical analysis in panels A, B, C, D and E. Data are presented as mean values +/- SEM. Related to figure 7. Source data are provided as a Source Data file.

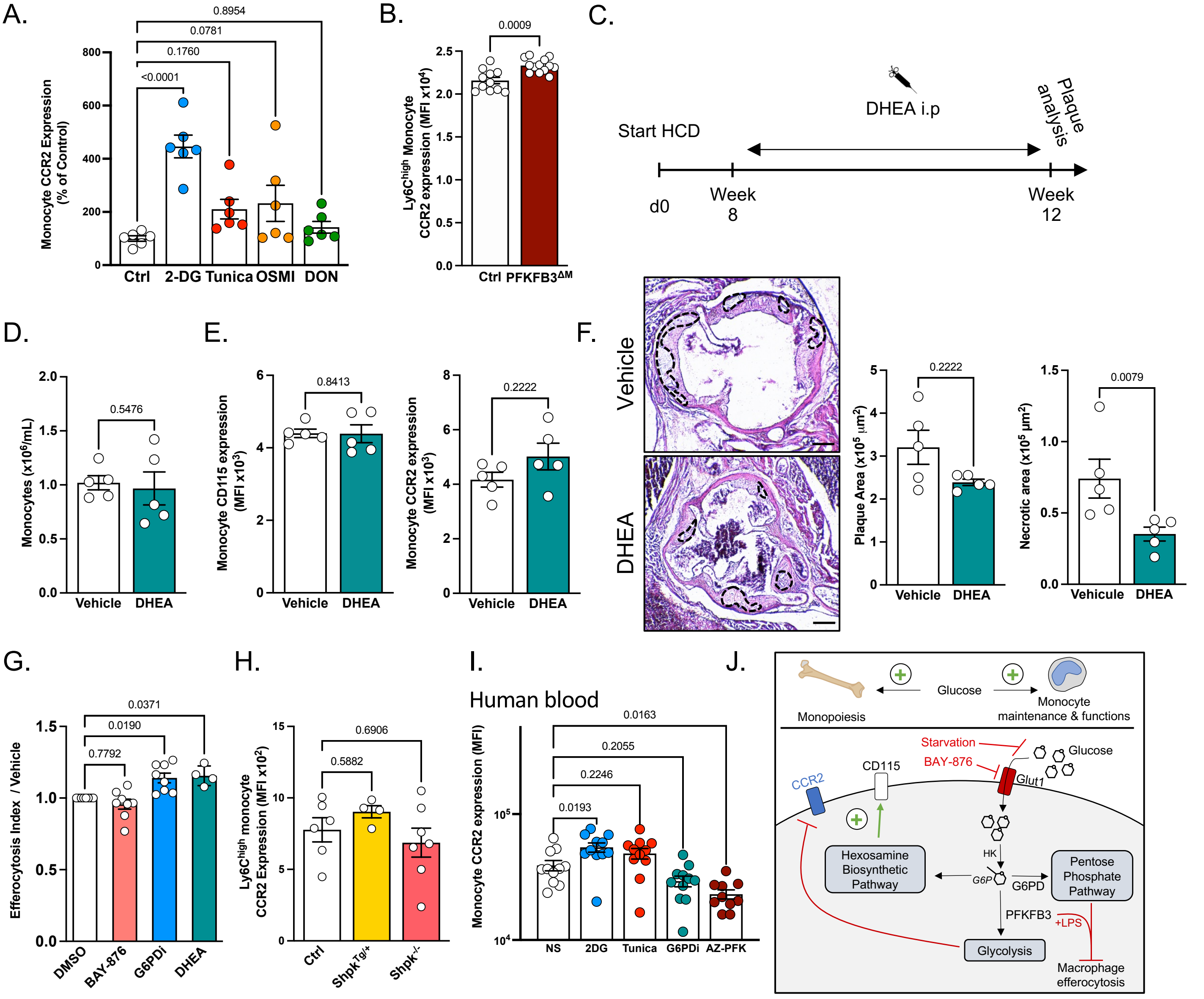

Supplementary figure 8. Impact of *in vivo* treatment with AZ-PFKFB3-67 and DHEA on atherosclerosis.
(**A**) Quantification of CCR2 expression on blood monocytes (from n=6 mice) incubated with vehicle, 2-DG, tunicamycin, OSMI-1 or DON. Data pooled from two independent experiments. (**B**) Quantification of surface CCR2 expression by blood monocytes from control (PFKFB3$^{fl/fl}$, n=11) and PFKFB3^{DM} (Lyz2$^{cre/+}$ x PFKFB3$^{fl/fl}$, n=13) mice. Data representative of 3 independent experiments. (**C**) Experimental scheme. LdlR$^{-/-}$ mice were fed western diet for 8 weeks before initiating a 4-week-long treatment with vehicle (n=5) DHEA (n=5) (7mg/kg/day). Data representative of two independent experiments. (**D**) Blood monocyte numbers in LdlR$^{-/-}$ mice after DHEA treatment. (**E**) Blood monocyte CD115 and CCR2 expression in LdlR$^{-/-}$ mice after DHEA treatment. (**F**) Quantification of plaque area and necrotic area in aortic arches from LdlR$^{-/-}$ mice after DHEA treatment, using H&E staining. Dotted lines indicate presence of necrotic zones. (**G**) Analysis of efferocytic capacity of large peritoneal macrophage treated with vehicle (DMSO, n=8), BAY-876 (n=8), G6PDi (n=8) or DHEA (n=4). Data pooled from two independent experiments. (**H**) Quantification of surface CCR2 expression by blood Ly6C^{high} monocytes from control (n=6), Shpk$^{Tg/+}$ (n=4) and Shpk$^{-/-}$ (n=7) mice. Data representative of 2 independent experiments. (**I**) Quantification of surface CCR2 expression on human blood monocytes (from n=11 healthy donors) treated with 2-DG, tunicamycin, G6PDi or AZ-PFKFB3-67. (**J**) Graphical summary of the findings reported in the study. One-way ANOVA with Dunnett's multplie comparisons tests were used for statistical analysis in panels A, G, H and I. Two-sided Mann-Whitney tests were used for statistical analysis in panels B, D, E, F. Data are presented as mean values +/- SEM. Related to figure 7. Source data are provided as a Source Data file.

Supplementary Table 1

Genotyping primers.

R26^{TdTomato}	5'AAGGGAGCTGCAGTGGAGTA3'	Wild-type forward primer
	5'CCGAAAATCTGTGGGAAGTC3'	Wild-type reverse primer
	5'CTGTTCCTGTACGGCATGG3'	Mutant forward primer
	5'GGCATTAAAGCAGCGTATCC3'	Mutant reverse primer
CX3CR1^{GFP}	5'GTCTTCACGTTCGGTCTGGT3'	Wild-type forward primer
	5'CCCAGACACTCGTTGTCCTT3'	Common reverse primer
	5'CTCCCCCTGAACCTGAAAC3'	Mutant forward primer
CCR2^{GFP}	5'AATAATCATTTTGTTCTCTGACCAC3'	Common forward primer
	5'ACAGCATGAACAATAGCCAAGT3'	Wild-type reverse primer
	5'CTGAACTTGTGGCCGTTTAC3'	Mutant reverse primer
LDLR$^{-/-}$	5'TATGCATCCCCAGTCTTTGG3'	Common forward primer
	5'CTACCCAACCAGCCCCTTAC3'	Wild-type reverse primer
	5'ATAGATTCGCCCTTGTGTCC3'	Mutant reverse primer
PFKFB3$^{fl/fl}$	5'CACCTGAGCAACATTGTAACT3'	Forward primer
	5'CAGGCCCAGACCAAGGACAGC3'	Reverse primer
Lyz2^{cre}	5'GTCACTCACTGCTCCCCTGT3'	Wild-type primer
	5'AAGGAGGGACTTGGAGGATG3'	Common primer
	5' ACCGGTAATGCAGGCAAAT3'	Mutant primer
Shpk$^{-/-}$	5'CCTCGTCACCTCTGCTTCTCTCC3'	Common forward primer
	5'CTTGGCCTGCTTTCCAAAATAGG3'	Wild-type reverse primer
	5'CACCTGCATTCCATCCCTAGACC3'	Mutant reverse primer
Shpk^{Tg}	5'GCTAACCATGTTCATGCCTTC3'	Forward primer
	5'GGGTGCAGCCTCCAATAG3'	Reverse primer
Csf1r$^{\Delta FIRE}$	5'GCTGCCCTGTCACTGTGTA3'	Forward primer
	5'TCGTTTCCCATCCCAGGA3'	Reverse primer

Supplementary Table 2

Key Resources Table

Reagent or resource	Source	Identifier
**<u>Antibodies</u>**		
α-mouse CD115 PE (clone AFS98)	Biolegend	Cat# 135506
α-mouse CD115 BV711 (clone AFS98)	Biolegend	Cat# 135515
α-mouse/human CD11b Brilliant Violet 510 (clone M1/70)	Biolegend	Cat# 101263
α-mouse/human CD11b PE-Cy5 (clone M1/70)	Biolegend	Cat# 101210
α-mouse/human CD11b BV750 (clone M1/70)	Biolegend	Cat# 101267
α-mouse Treml4 PE (clone 16E5)	Biolegend	Cat# 143304
α-mouse Gr1 PerCP-Cy5.5 (clone RB6-8C5)	BD Biosciences	Cat# 552093
α-mouse Gr1 FITC (clone RB6-8C5)	Biolegend	Cat# 108406
α-mouse CD16/32 PerCP-Cy5.5 (clone 93)	Biolegend	Cat# 156624
α-mouse CD34 BV421 (clone SA376A4)	Biolegend	Cat# 152208
α-mouse Ly6C BV421 (clone HK1.4)	Biolegend	Cat# 128032
α-mouse Ly6C BV711 (clone HK1.4)	Biolegend	Cat# 128037
α-mouse Ly6C BV605 (clone HK1.4)	Biolegend	Cat# 128036
α-mouse Ly6G BV785 (clone 1A8)	Biolegend	Cat# 127645
α-mouse ICAM2 AF647 (clone 3C4 (MIC2/4))	Biolegend	Cat# 105612
α-mouse CD9 APC-Fire750 (clone MZ3)	Biolegend	Cat# 124814
α-mouse CD3e BV605 (clone 145-2C11)	Biolegend	Cat# 100351
α-mouse CD3e APC (clone 145-2C11)	Biolegend	Cat# 100312
α-mouse CD4 AF700 (clone RM4-4)	Biolegend	Cat# 116022
α-mouse CD8 AF647 (clone 53-6.7)	Biolegend	Cat# 100724
α-mouse CD45 BV570 (clone 30-F11)	Biolegend	Cat# 103136
α-mouse F4/80 PE-Cy7 (clone BM8)	Biolegend	Cat# 123114
α-mouse CD45 APC-Cy7 (clone 30-F11)	BD Biosciences	Cat# 557659
α-mouse CD64 Brillant Violet 421 (clone X54-5/7.1)	Biolegend	Cat# 139309
α-mouse CD64 PE-Cy7 (clone X54-5/7.1)	Biolegend	Cat# 139314
α-mouse CD14 PE (clone M14-23)	Biolegend	Cat# 150106
α-mouse MerTK PE (clone 2B10C42)	Biolegend	Cat# 151506
α-mouse CD11c PE-Cy5 (clone N418)	Biolegend	Cat# 117316
α-mouse MHC-II (IA/IE) PB (clone M5/114.15.2)	Biolegend	Cat# 107620
α-mouse MHC-II (IA/IE) VioBlue (clone M5/114.15.2)	Miltenyi Biotec	Cat# 130-123-278
α-mouse CD226 BV421 (clone TX42.1)	Biolegend	Cat# 133615
α-mouse TCRβ PB (clone H57-597)	Biolegend	Cat# 109226
α-mouse NK1.1 APC (clone PK136)	Biolegend	Cat# 108720
α-mouse Ter119 APC (clone TER-119)	Biolegend	Cat# 116212
α-mouse B220 APC (clone RA3-6B2)	BD Biosciences	Cat# 561226
α-mouse CD19 BUV737 (clone 1D3)	BD Biosciences	Cat# 612781

α-mouse CD150 PE-Cy7 (clone TC15-12F12.2)	Biolegend	Cat# 115914
α-mouse Sca1 PB (clone D7)	Biolegend	Cat# 108120
α-mouse Sca1 PE-Cy7 (clone D7)	Biolegend	Cat# 108114
α-mouse c-Kit APC-Cy7 (clone ACK2)	eBioscience	Cat# 47-1172-82
α-mouse CD48 AF488 (clone HM48-1)	Biolegend	Cat# 103414
α-mouse CXCR4 APC (clone 2B11)	eBioscience	Cat# 51-9991-80
α-mouse CCR2 PE (clone REA538)	Miltenyi Biotec	Cat# 130-117-548
α-mouse CCR2 APC-Fire750 (clone SA203G11)	Biolegend	Cat# 150630
α-mouse CD11b APC (clone M1/70)	Biolegend	Cat# 101218
α-mouse Fc Block (clone 2.4G2)	BioXcell	Cat# BE0307
α-mouse Mac2	Cedarlane	Cat# CL8942AP
α-human CD45 APC-Cy7 (clone HI30)	Biolegend	Cat# 304014
α-human CD14 BV570 (clone M5E2)	Biolegend	Cat# 301832
α-human CCR2 APC (clone REA264)	Biolegend	Cat# 130-103-830
α-human CD115 PE (clone 9-4D2-1E4)	Biolegend	Cat# 347304
α-human CD88 PE-Dazzle594 (clone S5/1)	Biolegend	Cat# 344318
Anti-puromycin (clone 12D10)	Merck	Cat# MABE343
Anti-puromycin AF647	SCENITH kit	N/A
Cy™3 AffiniPure™ Mouse Anti-Rat IgG, Fcγ fragment specific	Jackson Immunoresearch	Cat# 212-165-104

Reagents

DAPI	Sigma	Cat# D9542
PFA 4%	VWR International	Cat# 9713.1000
Bovine serum Albumin (BSA)	Sigma	Cat# A7030
RPMI medium	Life Technologies	Cat# 21875091
DMEM medium	Life Technologies	Cat# 11960044
Collagenase A	Sigma	Cat# 11088793001
DNAse I	Sigma	Cat# 10104159001
Liberase	Roche	Cat# 5401054001
ImmunoHistoMount	Sigma	Cat# I1161
Fetal bovine serum	Fisher Scientific	Cat# 12350273
RBC lysing buffer	BD Biosciences	Cat# 555899
L-Glutamine	Life Technologies	Cat# 25030024
Penicillin Streptomycin	Life Technologies	Cat# 15070063
Sodium Pyruvate	Life Technologies	Cat# 11360039
Free glycerol reagent	Sigma	Cat# F6428
DreamTaq Green PCR Master Mix (2X)	Thermo Scientific	Cat# K1081
High cholesterol diet	Sniff	Cat# TD88137
BAY-876	MedChemExpress	Cat# HY-100017
DHEA	Sigma	Cat# 252805
G6PDi	MedChemExpress	Cat# HY-W107464
AZ-PFKFB3-67	MedChemExpress	Cat# HY-101972
Methylcellulose	Sigma Aldrich	Cat# M7027-100G
D-(+)-glucose powder	Sigma	Cat# G7021-100G
2-DG	Sigma	Cat# D6134-5G
Oligomycin A	Sigma	Cat# 75351-5MG
2-NBDG	Thermofisher	Cat# N13195

Tunicamycin	Thermo Scientific	Cat# J62217.MA
Puromycin	Merck	Cat# P7255
6-Diazo-5-oxo-L-nor-Leucine (DON)	MedChemExpress	Cat# HY-108357
OSMI-I	MedChemExpress	Cat# HY-119738
LPS	Sigma	Cat# L4391
Murine IL-4	Peprotech	Cat# 214-14
Brilliant Stain Buffer Plus	BD Biosciences	Cat# 566385
CellTrace™ Violet Cell Proliferation kit	Thermofisher	Cat# C34557
Phaseolus Vulgaris Leucoagglutinin (PHA-L), Biotinylated	Vector Labs	Cat# B-1115-2
Wheat Germ Agglutinin (WGA), Biotinylated	Vector Labs	Cat# B-1025-5
Sambucus Nigra Lectin (SNA, EBL), Biotinylated	Vector Labs	Cat# B-1305-2
Fluoresbrite® YG Microspheres 1μm	Polysciences	Cat# 17154
FluoSpheres™ Polystyrene Microspheres, 1.0 µm, red fluorescent	Invitrogen	Cat# F13083
EDTA	Sigma	Cat# 324504-500ML
Ficoll-PaqueTM PLUS	Cytiva	Cat # GE17-1440-02
Live/Dead fixable viability dye	Thermofisher	Cat #L34955
Zymosan A from Saccharomyces cerevisiae	Sigma	Cat# Z4250-250MG

Kits

CCL2 DuoSet ELISA	R&D Systems	Cat# DY479-05
TNF-alpha DuoSet ELISA	R&D Systems	Cat# DY410-05
NEFA-HR2 R1 + R2 FUJFILM	WAKO	Cat# W1W270-77000
Glucose dosage Kit	BioSentec	Cat# 075
Triglyceride dosage Kit	DiaSys	Cat# 157109910021
Foxp3 staining buffer	Miltenyi	Cat# 130-093-142
SCENITH kit	Dr. Rafael Arguello	N/A
PE/Cy7® Conjugation Kit - Lightning-Link®	Abcam	ab102903

Experimental models

Mouse: CX3CR1^{GFP} (B6.Cg-Ptprca Cx3cr1tm1Litt/LittJ)	The Jackson laboratory	Jax #008451
Mouse: Rosa26^{TdTomato} (B6.Cg-Gt(ROSA)26Sor$^{tm9(CAG-tdTomato)Hze}$/J)	The Jackson laboratory	Jax #007909
Mouse: CCR2^{GFP}	Dr. Marco Colonna	N/A
Mouse: Csf1r$^{\Delta FIRE}$	Dr. David Hume	N/A
Mouse: PFKFB3^{flox}	Dr. Peter Carmeliet	N/A
Mouse: C57BL/6J	The Jackson laboratory	Jax #000664
Mouse: Lyz2^{cre} (B6.129P2-Lyz2tm1(cre)Ifo/J)	The Jackson laboratory	Jax #004781
LdlR$^{-/-}$ (B6.129S7-Ldlrtm1Her/J)	The Jackson laboratory	Jax #002207
Mouse: Shpk^{Tg}	Dr. Arvand Haschemi	N/A
Mouse: Shpk$^{-/-}$ (RRID: MMRRC_043666-UCD)	Dr. Kent Lloyd	N/A

Accessories

StepOne	Applied Biosystem	N/A
Thermo Cycler SimpliAmp	Applied Biosystem	N/A
Nanodrop	OZYME	N/A
Veterinary hematology analyzer	Exigo	H400
Aurora 5 laser configuration	Cytek	N/A
BD FACS Canto II	BD Biosciences	N/A
	Mindray	N/A
Seahorse analyzer	Agilent Technologies	XF96

Softwares

Prism10	GraphPad	N/A
FlowJo	Tree Star	N/A
StepOne Software v.2.2.2	Applied Biosystem	N/A
Fiji	https://imagej.net/software/fiji/	N/A
