# Supplementary material for: Glucose metabolism controls monocyte homeostasis and migration but has no impact on atherosclerosis development in mice
Source: Nat Commun. 2024 Oct 19;15:9027. doi: 10.1038/s41467-024-53267-5 (PMC11489573; doi:10.1038/s41467-024-53267-5)
Supplement: Supplementary file 2 — Reporting Summary [file 41467_2024_53267_MOESM2_ESM.pdf]

nature portfolio

Corresponding author(s): Stoyan Ivanov, Alexandre Gallerand

Last updated by author(s): Sep 16, 2024

Reporting Summary

Nature Portfolio wishes to improve the reproducibility of the work that we publish. This form provides structure for consistency and transparency in reporting. For further information on Nature Portfolio policies, see our Editorial Policies and the Editorial Policy Checklist.

Statistics

For all statistical analyses, confirm that the following items are present in the figure legend, table legend, main text, or Methods section.

n/a	Confirmed	
[ ]	[x]	The exact sample size (n) for each experimental group/condition, given as a discrete number and unit of measurement
[ ]	[x]	A statement on whether measurements were taken from distinct samples or whether the same sample was measured repeatedly
[ ]	[x]	The statistical test(s) used AND whether they are one- or two-sided<br>*Only common tests should be described solely by name; describe more complex techniques in the Methods section.*
[x]	[ ]	A description of all covariates tested
[ ]	[x]	A description of any assumptions or corrections, such as tests of normality and adjustment for multiple comparisons
[ ]	[x]	A full description of the statistical parameters including central tendency (e.g. means) or other basic estimates (e.g. regression coefficient) AND variation (e.g. standard deviation) or associated estimates of uncertainty (e.g. confidence intervals)
[ ]	[x]	For null hypothesis testing, the test statistic (e.g. F, t, r) with confidence intervals, effect sizes, degrees of freedom and P value noted<br>*Give P values as exact values whenever suitable.*
[x]	[ ]	For Bayesian analysis, information on the choice of priors and Markov chain Monte Carlo settings
[x]	[ ]	For hierarchical and complex designs, identification of the appropriate level for tests and full reporting of outcomes
[x]	[ ]	Estimates of effect sizes (e.g. Cohen's d, Pearson's r), indicating how they were calculated

Our web collection on statistics for biologists contains articles on many of the points above.

Software and code

Policy information about availability of computer code

Data collection	Flow cytometry data were collected using a Cytek Aurora spectral flow cytometer with SpectroFlo software and a BD Canto II with Diva software.<br><br>For PET/CT, 45 to 60 minutes dynamic scan was performed after injection of 64Cu-DOTA984 ECL1i (3,7 MBq in 100µL saline) via tail vein with Inveon PET/CT system (Siemens, Malvern, 985 PA). PET images were reconstructed with the maximum a posteriori algorithm and analyzed by Inveon Research Workplace. Organs of interested were collected, weighed, and counted in a Beckman 8000 gamma counter (Beckman, Fullerton, CA). Radioactivities in the tissues were detected by autoradiograph using a Strom Phosphorimager (GE, Marlborough, MA).
Data analysis	Microsoft Excel.<br>Flowjo V10, Treestar.<br>Graphpad Prism 10.<br>Fiji analysis software V2.

For manuscripts utilizing custom algorithms or software that are central to the research but not yet described in published literature, software must be made available to editors and reviewers. We strongly encourage code deposition in a community repository (e.g. GitHub). See the Nature Portfolio guidelines for submitting code & software for further information.

Data

Policy information about availability of data

All manuscripts must include a data availability statement. This statement should provide the following information, where applicable:

- Accession codes, unique identifiers, or web links for publicly available datasets
- A description of any restrictions on data availability
- For clinical datasets or third party data, please ensure that the statement adheres to our policy

No new transcriptomic, proteomic or metabolomic datasets were generated during the study. All data supporting the findings of the study are presented in the main manuscript or in the supplementary information files. Source data are provided with this paper. Requests relative to the flow cytometry and imaging data presented herein should be directed to corresponding authors (Stoyan.Ivanov@univ-cotedazur.fr or Alexandre.Gallerand@univ-cotedazur.fr) due to still-ongoing analysis for further exploitation of the data. Access to our data shall be granted permanently to researchers who provide written research aims and are affiliated with a recognized institution. We aim to answer and provide access to requested data within 30 days.

Research involving human participants, their data, or biological material

Policy information about studies with human participants or human data. See also policy information about sex, gender (identity/presentation), and sexual orientation and race, ethnicity and racism.

Reporting on sex and gender	In accordance with the guidelines on sex and gender representation in human research participants, and to insure inclusivity of our findings, our study included a diverse sample of 11 participants, consisting of 6 females and 5 males. Data from male and female participants were pooled together as no sex-specific phenotypes were observed on the readouts analyzed.
Reporting on race, ethnicity, or other socially relevant groupings	Human blood donors were recruited solely on their will to participate in the study, and not on other social factors.
Population characteristics	Participants were 22 to 30 years-old, and healthy without any known conditions or current treatments.
Recruitment	Participants voluntered to donate blood after being informed about the goal of the study. All participants provided written informed consent.
Ethics oversight	Centre Hospitalier Universitaire de Nice.

Note that full information on the approval of the study protocol must also be provided in the manuscript.

Field-specific reporting

Please select the one below that is the best fit for your research. If you are not sure, read the appropriate sections before making your selection.

☒ Life sciences ☐ Behavioural & social sciences ☐ Ecological, evolutionary & environmental sciences

For a reference copy of the document with all sections, see nature.com/documents/nr-reporting-summary-flat.pdf

Life sciences study design

All studies must disclose on these points even when the disclosure is negative.

Sample size	No sample size calculations were performed. To comply with the principles of the 3Rs (Replacement, Reduction and Refinement), we have used minimal sample size sufficient to detect biological differences. Numbers of samples are indicated in the figure legends for each panel.
Data exclusions	We pre-established data exclusion criteria in order to avoid variance increases by outliers that would prevent us from drawing statistically meaningful conclusions, and creating the need for further experimentation on animal subjects. This initiative is in line withe the principle of the 3Rs. We determined that (1) mouse agressive behaviour and/or presence of fight wounds and (2) positivity to Grubb's test would be independent criteria for exclusion. Statistical outliers were identified using Grubb's test on the GraphPad Prism software.
Replication	The number of biologically-independent samples is indicated in figure legends corresponding to each panel.
Randomization	"Control" and "test" mouse groups were age-matched and sex-matched for in vivo experiment. Mice were randomly attributed to each experimental group when possible, depending on their genotype. Furthermore, if possible, animals were co-housed. In ex vivo experiments samples were also matched for age and sex. For in vitro experiments all allocations were random. Experimentators were blinded to group allocation during tissue collection and processing, and during acquisition of flow cytometry data. Experimentators were not blinded to group allocation during analysis of flow cytometry data. Experimentators were blinded to group allocation for all experiments linked to atherosclerotic plaque analysis, and data were uncoded by another experimentator.
Blinding	For tissue histology and cellular microscopy analyses, images were attributed a number by the experimentator (who was aware of the genotype and treatment used) and analyzed in a blind fashion by another member of the group who was unaware of the experimental parameters. Correspondance was revealed after image analysis. For other experiments (flow cytometry...), the experimentator was not blinded to the genotype and treatment as these informations were needed and known in order to start treatments and prepare the samples.

Reporting for specific materials, systems and methods

We require information from authors about some types of materials, experimental systems and methods used in many studies. Here, indicate whether each material, system or method listed is relevant to your study. If you are not sure if a list item applies to your research, read the appropriate section before selecting a response.

Materials & experimental systems

n/a	Involved in the study	
☐	☒	Antibodies
☒	☐	Eukaryotic cell lines
☒	☐	Palaeontology and archaeology
☐	☒	Animals and other organisms
☒	☐	Clinical data
☒	☐	Dual use research of concern
☒	☐	Plants

Methods

n/a	Involved in the study	
☒	☐	ChIP-seq
☐	☒	Flow cytometry
☒	☐	MRI-based neuroimaging

Antibodies

Antibodies used

a-mouse CD115 PE (clone AFS98) Biolegend Cat# 135506
a-mouse CD115 BV711 (clone AFS98) Biolegend Cat# 135515
a-mouse/human CD11b Brilliant Violet 510 (clone M1/70) Biolegend Cat# 101263
a-mouse/human CD11b PE-Cy5 (clone M1/70) Biolegend Cat# 101210
a-mouse/human CD11b BV750 (clone M1/70) Biolegend Cat# 101267
a-mouse Treml4 PE (clone 16E5) Biolegend Cat# 143304
a-mouse Gr1 PerCP-Cy5.5 (clone RB6-8C5) BD Biosciences Cat# 552093
a-mouse Gr1 FITC (clone RB6-8C5) Biolegend Cat# 108406
a-mouse CD16/32 PerCP-Cy5.5 (clone 93) Biolegend Cat# 156624
a-mouse CD34 BV421 (clone SA376A4) Biolegend Cat# 152208
a-mouse Ly6C BV421 (clone HK1.4) Biolegend Cat# 128032
a-mouse Ly6C BV711 (clone HK1.4) Biolegend Cat# 128037
a-mouse Ly6C BV605 (clone HK1.4) Biolegend Cat# 128036
a-mouse Ly6G BV785 (clone 1A8) Biolegend Cat# 127645
a-mouse ICAM2 AF647 (clone 3C4 (MIC2/4)) Biolegend Cat# 105612
a-mouse CD9 APC-Fire750 (clone MZ3) Biolegend Cat# 124814
a-mouse CD3e BV605 (clone 145-2C11) Biolegend Cat# 100351
a-mouse CD3e APC (clone 145-2C11) Biolegend Cat# 100312
a-mouse CD4 AF700 (clone RM4-4) Biolegend Cat# 116022
a-mouse CD8 AF647 (clone 53-6.7) Biolegend Cat# 100724
a-mouse CD45 BV570 (clone 30-F11) Biolegend Cat# 103136
a-mouse F4/80 PE-Cy7 (clone BM8) Biolegend Cat# 123114
a-mouse CD45 APC-Cy7 (clone 30-F11) BD Biosciences Cat# 557659
a-mouse CD64 Brillant Violet 421 (clone X54-5/7.1) Biolegend Cat# 139309
a-mouse CD64 PE-Cy7 (clone X54-5/7.1) Biolegend Cat# 139314
a-mouse CD14 PE (clone M14-23) Biolegend Cat# 150106
a-mouse MerTK PE (clone 2B10C42) Biolegend Cat# 151506
a-mouse CD11c PE-Cy5 (clone N418) Biolegend Cat# 117316
a-mouse MHC-II (IA/IE) PB (clone M5/114.15.2) Biolegend Cat# 107620
a-mouse MHC-II (IA/IE) VioBlue (clone M5/114.15.2) Miltenyi Biotec Cat# 130-123-278
a-mouse CD226 BV421 (clone TX42.1) Biolegend Cat# 133615
a-mouse TCR PB (clone H57-597) Biolegend Cat# 109226
a-mouse NK1.1 APC (clone PK136) Biolegend Cat# 108720
a-mouse Ter119 APC (clone TER-119) Biolegend Cat# 116212
a-mouse B220 APC (clone RA3-6B2) BD Biosciences Cat# 561226
a-mouse CD19 BUV737 (clone 1D3) BD Biosciences Cat# 612781
a-mouse CD150 PE-Cy7 (clone TC15-12F12.2) Biolegend Cat# 115914
a-mouse Sca1 PB (clone D7) Biolegend Cat# 108120
a-mouse Sca1 PE-Cy7 (clone D7) Biolegend Cat# 108114
a-mouse c-Kit APC-Cy7 (clone ACK2) eBioscience Cat# 47-1172-82
a-mouse CD48 AF488 (clone HM48-1) Biolegend Cat# 103414
a-mouse CXCR4 APC (clone 2B11) eBioscience Cat# 51-9991-80
a-mouse CCR2 PE (clone REA538) Miltenyi Biotec Cat# 130-117-548
a-mouse CCR2 APC-Fire750 (clone SA203G11) Biolegend Cat# 150630
a-mouse CD11b APC (clone M1/70) Biolegend Cat# 101218
a-mouse Fc Block (clone 2.4G2) BioXcell Cat# BE0307

a-mouse Mac2 Cedarlane Cat# CL8942AP
a-human CD45 APC-Cy7 (clone HI30) Biolegend Cat# 304014
a-human CD14 BV570 (clone M5E2) Biolegend Cat# 301832
a-human CCR2 APC (clone REA264) Biolegend Cat# 130-103-830
a-human CD115 PE (clone 9-4D2-1E4) Biolegend Cat# 347304
a-human CD88 PE-Dazzle594 (clone S5/1) Biolegend Cat# 344318
Anti-puromycin (clone 12D10) Merck Cat# MABE343
Anti-puromycin AF647 SCENITH kit N/A
Cy™3 AffiniPure™ Mouse Anti-Rat IgG, Fcγ fragment specific Jackson Immunoresearch Cat# 212-165-104

Validation

All antibodies were obtained from commercial sources and validation data as well as detailed information can be found on the manufacturer's website.

Animals and other research organisms

Policy information about studies involving animals; ARRIVE guidelines recommended for reporting animal research, and Sex and Gender in Research

Laboratory animals

Wild-type C57BL/6J (Jax #000664), CX3CR1gfp (B6.Cg-Ptprca Cx3cr1tm1Litt/LittJ, Jax # #008451), Lyz2cre (B6.129P2-Lyz2tm1(cre)Ifo/J, Jax #004781), R26TdTomato (B6.Cg-Gt(ROSA)26Sortm9(CAG-tdTomato)Hze/J, Jax #007909), and LdlR-/- (B6.129S7-Ldlrtm1Her/J, Jax #002207) mice used in this study were maintained under C57BL/6J background and originally purchased from Janvier Labs. PFKFB3flox mice49 were kindly provided by Dr. Peter Carmeliet and crossed to Lyz2cre mice. Shpk mouse strains were in a C57BL/6N background and heterozygote Shpk over-expressing transgenic mice (ShpkTg/+) were developed in the Haschemi Lab. For this purpose, mouse Shpk mRNA coding sequence (NM_029031.3) was cloned into pCAGGS plasmid52 (GenBank: LT727518.1, kindly provided by the BCCM/LMBP Plasmid collection) and sequence integrity was verified by DNA sequencing. The transgene (insert) was generated using Sal I and HIND III restriction sites with a total size of 3.7 kB, including regulatory elements. Transgenic animals were successfully generated with the help of Thomas Rülicke (University of Veterinary Medicine Vienna) and Biomodels Austria by pronuclear transgene microinjection using fertilized mouse embryos of the C57BL/6N background. The Shpk-/- mouse strain (RRID: MMRRC_043666-UCD) was obtained from the MMRRC at University of California at Davis and was donated by Kent Lloyd, D.V.M., University of California, Davis. CCR2GFP mice37 (B6(C)-Ccr2tm1.1Cln/J, Jax #027619) were provided by Dr. Marco Colonna. Csf1rFIRE mice38 were provided by Dr. David Hume to Dr. Marc Bajénoff, and both researchers kindly agreed to share the mice with our group. Experimental and control animals were co-housed, and littermate controls were used as often as possible. Animals of mixed sex and similar age (7 to 12 weeks old) were used within each cohort. Since we did not observe any sex-specific phenotypes, we decided to group data from male and female mice together in experiments where mice of both sexes were used. All mice were bred and housed in specific pathogen-free conditions maintained in facilities in the Mediterranean Center of Molecular Medicine (INSERM U1065, Université Côte d'Azur), Washington University in Saint Louis animal facility, the University of Minnesota Medical School Research Animal Resources facility, the Medical University of Vienna animal facility or the Centre d'Immunologie de Marseille Luminy facility. An ambient temperature of ~20-23°C was maintained, with a 12/12-hour light/dark cycle and food available ad libitum. Animals were euthanized by cervical dislocation.

Wild animals

No wild animals were used in the study.

Reporting on sex

Animals of mixed sex and similar age were used within each cohort. Since we did not observe any sex-specific phenotypes, we decided to group data from male and female mice together in experiments where mice of both sexes were used.

Field-collected samples

No field-collected samples were used in the study.

Ethics oversight

Animal protocols required for experimentation other than organ collection were authorized by the French Ministry of Higher Education and Research upon approval of the local ethical committee (CIEPAL Azur) at Université Côte d'Azur, and by the Institutional Animal Care and Use Committee (IACUC) at Washington University in Saint Louis and University of Minnesota Medical School.

Note that full information on the approval of the study protocol must also be provided in the manuscript.

Plants

Seed stocks

Report on the source of all seed stocks or other plant material used. If applicable, state the seed stock centre and catalogue number. If plant specimens were collected from the field, describe the collection location, date and sampling procedures.

Novel plant genotypes

Describe the methods by which all novel plant genotypes were produced. This includes those generated by transgenic approaches, gene editing, chemical/radiation-based mutagenesis and hybridization. For transgenic lines, describe the transformation method, the number of independent lines analyzed and the generation upon which experiments were performed. For gene-edited lines, describe the editor used, the endogenous sequence targeted for editing, the targeting guide RNA sequence (if applicable) and how the editor was applied.

Authentication

Describe any authentication procedures for each seed stock used or novel genotype generated. Describe any experiments used to assess the effect of a mutation and, where applicable, how potential secondary effects (e.g. second site T-DNA insertions, mosiacism, off-target gene editing) were examined.

Flow Cytometry

Plots

Confirm that:

- [x] The axis labels state the marker and fluorochrome used (e.g. CD4-FITC).
- [x] The axis scales are clearly visible. Include numbers along axes only for bottom left plot of group (a 'group' is an analysis of identical markers).
- [x] All plots are contour plots with outliers or pseudocolor plots.
- [x] A numerical value for number of cells or percentage (with statistics) is provided.

Methodology

Sample preparation	Tissues were harvested after cervical dislocation and washed in PBS. Splenocytes were prepared by gently crushing the spleen on a 70m strainer in flow buffer (PBS containing 1% BSA and 2mM EDTA). Peritoneal cells were obtained by performing lavage with 5mL flow buffer. Aortas were washed and carefully dissected to remove surrounding adipose tissue, before being digested using 100g/mL DNAse I and 300ug/mL LiberaseTM TL (Roche). Bone marrow cells were prepared by flushing femurs and tibias with flow buffer. Blood was drawn from the submandibular vein and collected in heparinized tubes or Eppendorf tubes containing 15l 500mM EDTA. Leukocytes were counted using a veterinary hematology analyzer (Exigo H400). Red blood cells were lysed from all single cell suspensions using BD Pharm Lyse lysing solution (BdBiosciences cat #555899). Cells were washed with PBS, stained with violet Live/Dead fixable viability dye (Thermofisher cat #L34955), washed in flow buffer and then stained. For intracellular staining, cells were fixed and permeabilized using Miltenyi Foxp3 staining buffer (cat #130-093-142). For CCR2 staining in Figure 6I, blood was collected in heparin tubes and then incubated at 37°C for the indicated time before staining. All antibodies were used 1/200. A list of all antibodies used in provided in Supplementary Table 2. Flow cytometry data were acquired using a BD FACS Canto II and a Cytek Aurora cytometer with 5 laser configuration. All analyses, including unsupervised t-SNE analysis, were performed using FlowJo software (Tree Star).
Instrument	BD CANTO II, Cytek Aurora
Software	FACS DIVA<br>SpectroFlo<br>Flowjo
Cell population abundance	Cell sorting was not used in the study.
Gating strategy	Gating strategies are indicated in dedicated panels or supplementary figures throughout the manuscript.

- [x] Tick this box to confirm that a figure exemplifying the gating strategy is provided in the Supplementary Information.
