# Supplementary material for: Glucose metabolism controls monocyte homeostasis and migration but has no impact on atherosclerosis development in mice
Source: Nat Commun. 2024 Oct 19;15:9027. doi: 10.1038/s41467-024-53267-5 (PMC11489573; doi:10.1038/s41467-024-53267-5)
Supplement: Supplementary file 3 — Source Data [file 41467_2024_53267_MOESM3_ESM.pdf]

nature portfolio

Peer Review File

Glucose metabolism controls monocyte homeostasis and migration but has no impact on atherosclerosis development in mice.

Corresponding Author: Dr Stoyan Ivanov

This manuscript has been previously reviewed at another journal. This document only contains reviewer comments, rebuttal for versions considered at Nature Communications.

This file contains all reviewer reports in order by version, followed by all author rebuttals in order by version.

Version 1:

Reviewer comments:

Reviewer #1

(Remarks to the Author)
The authors were quite responsive.

We disagree on the value of in vitro experiments; I maintain my view (which is shared by many macrophage experts) that in vitro experiments are often not reflective of in vivo processes. The authors should offer a limitation statement on this point in the manuscript.

The addition of FACS data is an advance.

The response regarding the cre are reasonable but should also be mentioned in the manuscript.

I have trouble following the attempt to reconcile prior work on CCR2 and the current findings.

Reviewer #2

(Remarks to the Author)
The authors responded to all of my comments and suggestions. Most of them are adequately addressed, but not all. Tw comments need additional attention.

First, in line 219 of the Rebuttal, I mentioned the caveat that GLUT1 is expressed on many cells, and not myeloid cells, such that from the results of administration BAY-876 it is impossible to draw conclusions on glucose uptake specifically in myeloid cells. The authors responded by showing that there are no systemic effects on glucose, cholesterol, etc, but further they only added one sentence in the Discussion lines 632 ‘Nevertheless, we cannot exclude that BAY-876 treatment might impact other immune or stromal cells’. I think it is very likely this also affected other cell functions, e.g. endothelial cells, which highly depend on glycolysis, which is further upregulated in atherosclerosis-prone regions with disturbed flow. So I think this limitations warrants a more extensive discussion in the limitations section.

Secondly, in my comment in line 279 of the Rebuttal I mentioned ‘the authors suggest that the similar recruitment of monocytes after Glut1 inhibition despite lower monocyte levels is due to upregulation of CCR2, however this is not experimentally proven. It would be critical to establish this causal role to justify the final conclusion of the Discussion in line 555 that reducing CCR2-dependent migration could potential improve CVD outcome in patients with diabetes.’ The authors respond that they have proven that glucose flux affects CCR2 expression. However, this was not wat I meant. Indeed, they have proven that there is an upregulation of CCR2 expression on the remaining monocytes in the circulation, but they did not proof that this increased expression caused the increased recruitment rate. Other mechanisms, e.g. on endothelial cells, could in theory also contribute. I think this should be discussed at the future perspective of lines 646-648 should be toned

down.

Reviewer #4

(Remarks to the Author)
Gallerand et al., explored how circulating metabolites influence monocyte behavior. Glucose metabolism is crucial for maintaining monocyte homeostasis, membrane expression of CD115, and migratory capacity via CCR2 expression; however, atherosclerotic plaque development was unaffected, as remaining monocytes exhibited enhanced migratory capacity.

Undoubtedly, the preclinical and in vitro data presented here and in earlier studies are beginning to indicate that glucose metabolism is crucial for coordinated immunomodulatory effects to maintain immune homeostasis.

Despite the message of the findings, this reviewer pointed out that the study by Gallerand et al., still lacks substantial novel insights and conceptual advancement, as it reiterates known phenomena about the relationship between glucose metabolism and monocyte behavior without providing deeper understanding or strategic follow-up on both positive and negative findings. Therefore, although the experimental techniques and overall technical quality of the paper are outstanding, it is reasonable to concern the issue of novelty. The key concepts revealed in this paper have already been reported in previous studies (Jordan S et al., Cell 2019, Collins et al., Cell 2019, Nagai et al., Cell 2019).

Reviewer #5

(Remarks to the Author)
I co-reviewed this manuscript with one of the reviewers who provided the listed reports. This is part of the Nature Communications initiative to facilitate training in peer review and to provide appropriate recognition for Early Career Researchers who co-review manuscripts.

Version 2:

Reviewer comments:

Reviewer #1

(Remarks to the Author)
no further comments

Reviewer #2

(Remarks to the Author)
With the addition of a Study Limitations paragraph including the lack of myeloid specificity of Glut1 inhibition, the authors adequately responded to my last comments.
I do not have any further comments.

Reviewer #4

(Remarks to the Author)
The authors did not spare any efforts to revise the manuscript.
This reviewer agrees to the publication of this manuscript in Nature Communications.

Reviewer #5

(Remarks to the Author)
I co-reviewed this manuscript with one of the reviewers who provided the listed reports. This is part of the Nature Communications initiative to facilitate training in peer review and to provide appropriate recognition for Early Career Researchers who co-review manuscripts.

Open Access This Peer Review File is licensed under a Creative Commons Attribution 4.0 International License, which permits use, sharing, adaptation, distribution and reproduction in any medium or format, as long as you give appropriate credit to the original author(s) and the source, provide a link to the Creative Commons license, and indicate if changes were made.
In cases where reviewers are anonymous, credit should be given to 'Anonymous Referee' and the source.
The images or other third party material in this Peer Review File are included in the article’s Creative Commons license, unless indicated otherwise in a credit line to the material. If material is not included in the article’s Creative Commons license and your intended use is not permitted by statutory regulation or exceeds the permitted use, you will need to obtain permission directly from the copyright holder.
To view a copy of this license, visit https://creativecommons.org/licenses/by/4.0/

REVIEWER COMMENTS

Reviewer #1 (Remarks to the Author):

The authors were quite responsive.
We thank the reviewer for her/his careful evaluation of our revised manuscript.

We disagree on the value of in vitro experiments; I maintain my view (which is shared by many macrophage experts) that in vitro experiments are often not reflective of in vivo processes. The authors should offer a limitation statement on this point in the manuscript.
We agree with the reviewer that the value of in vitro experiments is inferior to that of in vivo experiments, especially in tissue resident macrophage biology which is highly dependent on microenvironment. We included a statement in the study limitations section to clarify this point (p.16l.667).

The addition of FACS data is an advance.
As indicated in our previous response, we thank the reviewer for suggesting to use flow cytometry which yielded important results.

The response regarding the cre are reasonable but should also be mentioned in the manuscript.
We have added a statement in the study limitations section about the potential caveats of the cre used (p.16l.672).

I have trouble following the attempt to reconcile prior work on CCR2 and the current findings.
Blood monocyte counts positively correlate with atherosclerosis development in humans[1,2]. Prior work on murine models established the importance of CCR2-dependent chemotaxis in monocyte biology and atherosclerosis progression. The key paper indicated by the reviewer showed reduced atherosclerosis development in mice lacking CCR2[3], a phenotype directly linked to the requirement of CCR2 for recruitment to inflammatory sites[4,5]. However, CCR2-deficient mice also show strongly reduced blood monocyte counts as these cells are retained in bone marrow[5], and thus do not recapitulate human disease (high blood monocyte counts with functional CCR2). Furthermore, plaque formation is reduced but not abolished in CCR2-deficient mice[3]. This could be due to recruitment of (few remaining) monocytes via other chemokine receptors[6]. It is challenging to uncouple the effects of reduced monocyte counts versus their lack of CCR2 in CCR2-KO mice. When we first observed reduction of blood monocyte counts upon BAY-876 treatment, we hypothesized that plaque development would be halted by the treatment based on work cited above. However, this was not the case as shown in the manuscript, since the migratory capacity of remaining monocytes was increased. Our study is thus complementary rather than contradictory with prior key studies. Indeed, we found that monocyte migration capacity is an important risk factor (as demonstrated for high blood monocyte numbers), and could be considered as a valuable readout in humans presenting plaque-associated risk-factors but not high monocyte counts.

Reviewer #2 (Remarks to the Author):

The authors responded to all of my comments and suggestions. Most of them are adequately addressed, but not all. Tw comments need additional attention.
We thank the reviewer for her/his careful evaluation of our revised manuscript and hope to clarify remaining points.

First, in line 219 of the Rebuttal, I mentioned the caveat that GLUT1 is expressed on many cells, and not myeloid cells, such that from the results of administration BAY-876 it is impossible to draw conclusions on glucose uptake specifically in myeloid cells. The authors responded by showing that there are no systemic effects on glucose, cholesterol, etc, but further they only added one sentence in the Discussion lines 632 'Nevertheless, we cannot exclude that BAY-876 treatment might impact other immune or stromal cells'. I think it is very likely this also affected other cell functions, e.g. endothelial cells, which highly depend on glycolysis, which is further upregulated in atherosclerosis-prone regions with disturbed flow. So I think this limitations warrants a more extensive discussion in the limitations section.

We agree with the reviewer that the lack of selective effects of BAY-876 treatment should be further emphasized. We included additional information on this matter (p.15l.655).

Secondly, in my comment in line 279 of the Rebuttal I mentioned ‘the authors suggest that the similar recruitment of monocytes after Glut1 inhibition despite lower monocyte levels is due to upregulation of CCR2, however this is not experimentally proven. It would be critical to establish this causal role to justify the final conclusion of the Discussion in line 555 that reducing CCR2-dependent migration could potential improve CVD outcome in patients with diabetes.’ The authors respond that they have proven that glucose flux affects CCR2 expression. However, this was not wat I meant. Indeed, they have proven that there is an upregulation of CCR2 expression on the remaining monocytes in the circulation, but they did not proof that this increased expression caused the increased recruitment rate. Other mechanisms, e.g. on endothelial cells, could in theory also contribute. I think this should be discussed at the future perspective of lines 646-648 should be toned down.
We thank the reviewer for this clarification. We modified our future perspectives (p.15l.650) and discussed the limitations relative to endothelial cell functions in our study limitations section (p.15l.657).

Reviewer #4 (Remarks to the Author):

Gallerand et al., explored how circulating metabolites influence monocyte behavior. Glucose metabolism is crucial for maintaining monocyte homeostasis, membrane expression of expression; however, atherosclerotic plaque development was unaffected, as remaining monocytes exhibited enhanced migratory capacity.

Undoubtedly, the preclinical and in vitro data presented here and in earlier studies are beginning to indicate that glucose metabolism is crucial for coordinated immunomodulatory effects to maintain immune homeostasis.

Despite the message of the findings, this reviewer pointed out that the study by Gallerand et al., still lacks substantial novel insights and conceptual advancement, as it reiterates known phenomena about the relationship between glucose metabolism and monocyte behavior without providing deeper understanding or strategic follow-up on both positive and negative findings. Therefore, although the experimental techniques and overall technical quality of the paper are outstanding, it is reasonable to concern the issue of novelty. The key concepts revealed in this paper have already been reported in previous studies (Jordan S et al., Cell 2019, Collins et al., Cell 2019, Nagai et al., Cell 2019).
We thank the reviewer for her/his evaluation of our revised manuscript. We agree that the previous studies cited by the reviewer outlined important phenomena linking glucose metabolism and monocyte biology. Our study is very complimentary with previous literature, as we uncover phenotypes that were not described previously and further enhance our understanding of how glucose metabolism regulates monocyte biology. While we confirm a key role of glucose on maintenance of the blood monocyte pool, we further dissect this phenomenon by looking at monocyte intracellular metabolism as well as monocyte generation in the bone marrow. Furthermore, we report that glucose metabolism controls monocyte CD115 and CCR2 expression, as well as their recruitment to tissues and their trajectories of differentiation to macrophages in those tissues through defined intracellular pathways. On a pathophysiological standpoint, we – very surprisingly – report that strongly reducing monocyte counts in atherosclerosis-prone mice by inhibiting Glut1 does not affect plaque size and phenotype, since glucose metabolism also controls monocyte migratory capacity.

We are now deciphering how the phenotypes observed (differential generation, survival, recruitment and differentiation upon Glut1 inhibition) might directly link to alterations of CD115 signaling. CSF1 binding is known to promote glycolysis in monocyte-derived macrophages[7-9]. This reprogramming depends on the CSF1R-AKT-mTOR pathway[7], and we observed reduced AKT and mTOR phosphorylation in starved monocytes (Figure 2A). We thus asked how CD115 signaling might control intracellular monocyte metabolism. We treated mice with AFS98 (CD115 blocking antibody) and then performed SCENITH on blood monocytes (Reply Figure 1A). Monocyte glycolytic capacity was reduced while their mitochondrial dependence increased following CD115 blockade (Reply Figure 1B). However, blood monocyte proportions were not affected by CD115 blockade (Reply Figure 1C). We next explored the impact of CD115 blockade during starvation challenge (Reply Figure 1D). Ly6C^{high} blood monocyte counts were still unaffected, however Ly6C^{low} monocyte numbers tended to be diminished (Reply Figure 1E). This was associated with diminution of AKT phosphorylation in Ly6C^{low} but not Ly6C^{high} monocytes (Reply Figure 1F). These results indicate that CD115 signaling promotes glucose intracellular utilization in monocytes. In starved conditions, where glucose is scarce and CD115 is downregulated, blocking signaling of remaining CD115 protein triggers loss of Ly6C^{low}

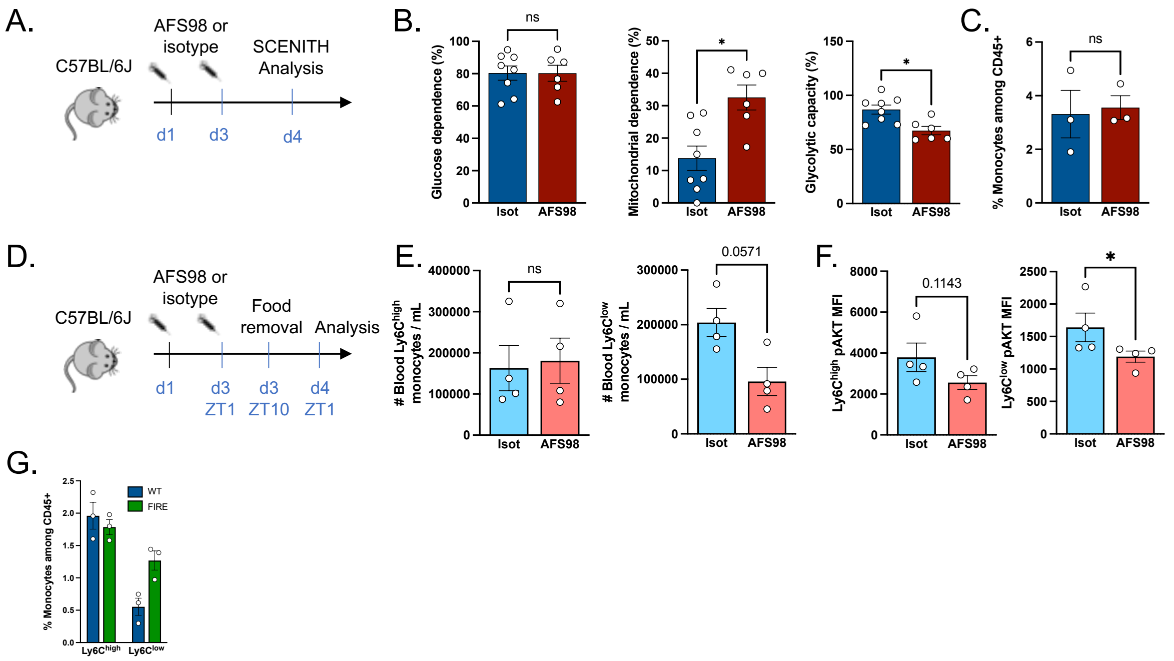

Figure 1. Impact of CD115 blockade on monocyte metabolism.
A. Experimental scheme used to evaluate impact of CD115 blockade on monocyte metabolism. B. Monocyte metabolic parameters measured by SCENITH following CD115 blockade. C. Blood monocyte proportions following treatment with isotype control or AFS98. D. Experimental scheme used to test impact of concomitant starvation challenge and CD115 blockade on blood monocytes. E-F. Quantification of blood monocyte subsets (E) and AKT phosphorylation (F) following treatment with isotype control or AFS98 and starvation challenge. G. Proportions of monocyte subsets in blood from control (WT) and FIRE mice at steady state.

monocytes from blood (Reply Figure 1E). Curiously, Ly6C^{low} monocytes are more abundant in blood from Csf1r$^{\Delta FIRE}$ (FIRE) mice compared to controls (Reply Figure 1G), despite their complete loss of CD115 expression[10], indicating that CD115 is not required for monocyte survival. Whether the phenotypes reported herein (Reply Figure 1D-F) reflect reduced differentiation from Ly6C^{high} monocytes, reduced viability or augmented adherence to endothelium is currently under investigation. Due to their limited numbers, robust analysis of labelled glucose utilization in metabolic pathways in blood monocytes is challenging. Thus, one could wonder how precisely glucose flux is rewired in monocytes at steady-state, during starvation and in condition of excessive plasma glucose.

Reviewer #5 (Remarks to the Author):

I co-reviewed this manuscript with one of the reviewers who provided the listed reports. This is part of the Nature Communications initiative to facilitate training in peer review and to provide appropriate

recognition for Early Career Researchers who co-review manuscripts.
We thank the co-reviewer for her/his evaluation of our revised manuscript.

1 Chapman, C. M., Beilby, J. P., McQuillan, B. M., Thompson, P. L. & Hung, J. Monocyte count, but not C-reactive protein or interleukin-6, is an independent risk marker for subclinical carotid atherosclerosis. *Stroke* **35**, 1619-1624, doi:10.1161/01.STR.0000130857.19423.ad (2004).

2 Johnsen, S. H. *et al.* Monocyte count is a predictor of novel plaque formation: a 7-year follow-up study of 2610 persons without carotid plaque at baseline the Tromso Study. *Stroke* **36**, 715-719, doi:10.1161/01.STR.0000158909.07634.83 (2005).

3 Boring, L., Gosling, J., Cleary, M. & Charo, I. F. Decreased lesion formation in CCR2-/- mice reveals a role for chemokines in the initiation of atherosclerosis. *Nature* **394**, 894-897, doi:10.1038/29788 (1998).

4 Boring, L. *et al.* Impaired monocyte migration and reduced type 1 (Th1) cytokine responses in C-C chemokine receptor 2 knockout mice. *J. Clin. Invest.* **100**, 2552-2561, doi:10.1172/JCI119798 (1997).

5 Tsou, C. L. *et al.* Critical roles for CCR2 and MCP-3 in monocyte mobilization from bone marrow and recruitment to inflammatory sites. *J. Clin. Invest.* **117**, 902-909, doi:10.1172/JCI29919 (2007).

6 Tacke, F. *et al.* Monocyte subsets differentially employ CCR2, CCR5, and CX3CR1 to accumulate within atherosclerotic plaques. *J. Clin. Invest.* **117**, 185-194, doi:10.1172/JCI28549 (2007).

7 Chang, M. *et al.* Phosphatidylinostitol-3 kinase and phospholipase C enhance CSF-1-dependent macrophage survival by controlling glucose uptake. *Cell. Signal.* **21**, 1361-1369, doi:10.1016/j.cellsig.2009.04.003 (2009).

8 Hamilton, J. A., Vairo, G. & Lingelbach, S. R. CSF-1 stimulates glucose uptake in murine bone marrow-derived macrophages. *Biochem Biophys Res Commun* **138**, 445-454, doi:10.1016/0006-291x(86)90301-3 (1986).

9 Hamilton, J. A., Vairo, G. & Lingelbach, S. R. Activation and proliferation signals in murine macrophages: stimulation of glucose uptake by hemopoietic growth factors and other agents. *J. Cell. Physiol.* **134**, 405-412, doi:10.1002/jcp.1041340311 (1988).

10 Rojo, R. *et al.* Deletion of a Csf1r enhancer selectively impacts CSF1R expression and development of tissue macrophage populations. *Nat Commun* **10**, 3215, doi:10.1038/s41467-019-11053-8 (2019).

REVIEWERS' COMMENTS

Reviewer #1 (Remarks to the Author):
no further comments

Reviewer #2 (Remarks to the Author):
With the addition of a Study Limitations paragraph including the lack of myeloid specificity of Glut1 inhibition, the authors adequately responded to my last comments.
I do not have any further comments.

Reviewer #4 (Remarks to the Author):
The authors did not spare any efforts to revise the manuscript.
This reviewer agrees to the publication of this manuscript in Nature Communications.

Reviewer #5 (Remarks to the Author):
I co-reviewed this manuscript with one of the reviewers who provided the listed reports. This is part of the Nature Communications initiative to facilitate training in peer review and to provide appropriate recognition for Early Career Researchers who co-review manuscripts.

We would like to thank all reviewers for their careful evaluation of our manuscript, their feedback and their comments which we believe helped us improve the manuscript.
